# Schottky Interface Enabled Electrospun Rhodium Oxide Doped Gold for Both pH Sensing and Glucose Measurements in Neutral Buffer and Human Serum

**DOI:** 10.1021/acs.langmuir.4c02999

**Published:** 2024-09-17

**Authors:** Weiyu Xiao, Mingman Li, Danlei Li, Bo Shi, Runze Zhong, Yiyuan Zhao, Qingliang Tai, Songbing He, Qiuchen Dong

**Affiliations:** † Department of Chemistry, School of Science, Xi′an Jiaotong-Liverpool University, No. 111 Ren’ai Road, Suzhou Industrial Park, Dushu Lake Higher Education and Innovation Park, Suzhou 215123, Jiangsu Province, People’s Republic of China; ‡ Department of General Surgery, First Affiliated Hospital of Soochow University, No. 188 Shizi Street, Suzhou 215006, Jiangsu Province, People’s Republic of China

## Abstract

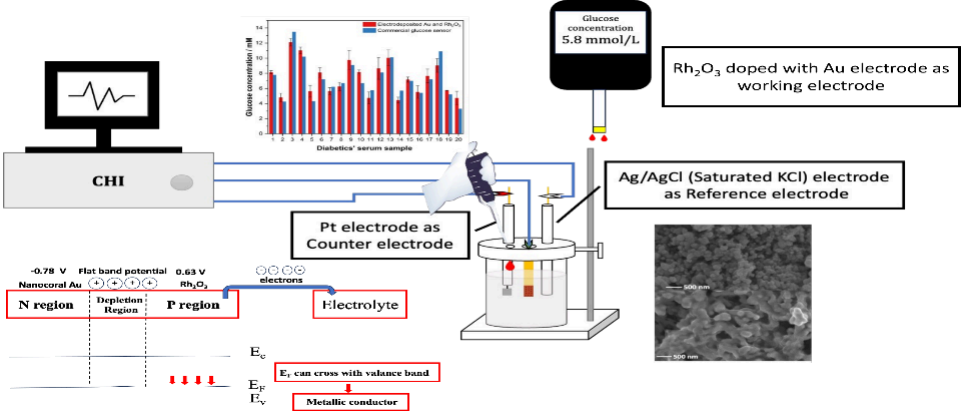

This study has focused on adjusting sensing environment from basic to neutral pH and improve sensing performance by doping electrodeposited gold (Au) with metal oxide for nonenzymatic glucose measurements in forming a Schottky interface for superior glucose sensing with detailed analysis for the sensing mechanism. The prepared sensor also holds the ability to measure pH with the identical electrospun metal oxide-electrodeposited Au, which composed a dual sensor (glucose and pH sensor) through applying chronoamperometry and open circuit potential methods. The rhodium oxide nanocoral structure was fabricated with an electrospinning precursor solution, followed by a calcination process, and it was mixed with electrodeposited nanocoral gold to form the Schottky interface by constructing a p-n type heterogeneous junction for improved sensitivity in glucose detection. The prepared materials were characterized by X-ray powder diffraction (XRD), scanning electron microscopy (SEM), transmission electron microscopy (TEM), and X-ray photoelectron spectrometry (XPS), etc. The prepared materials were used for both pH responsive testing and amperometric glucose measurements. The rhodium oxide nanocoral doped gold demonstrated a sensitivity of 3.52 μA mM^–1^ cm^–2^ and limit of detection of 20 μM with linear range up to 3 mM glucose concentration compared to solely electrodeposited gold for a sensitivity of 0.46 μA mM^–1^ cm^–2^ and a limit of detection of 450 μM. The Mott–Schottky method was used for the analysis of an electron transfer process from noble metal to metal oxide to electrolyte in demonstrating the improved sensitivity at neutral pH for glucose measurements due to the Schottky barrier adjustment mechanism at an applied flat band potential of 0.3 V. This work opens a new venue in illustrating the metal oxide/metal materials in the glucose neutral response mechanism. In the end, human serum samples were tested against current commercial glucose meter to certify the accuracy of the proposed sensor.

## Introduction

Type (I) and Type II are two types of commonly known diabetes categories. Type (I) diabetes is not preventable due to the lack of insulin generation by pancreatic islet cells.^1^ A person who lives with a condition of undiagnosed and untreated diabetes will suffer a prolonged and worse health outcome. Thus, it is important to have basic diagnostics to the fluctuated blood glucose level with accurate monitoring for preventing development into severe complications. Currently, glucose monitoring is mainly managed by intermittent and continuous monitoring. However, both methods rely on using the glucose dehydrogenase or glucose oxidase for the oxidation of glucose molecules in capturing the electrochemical signal in such a process.^2^ The enzyme-based electrochemical sensors suffer from unstable performance and shortened storage time due to the fluctuated local pH and temperature.^3,4^

The current available nonenzymatic glucose electrochemical sensors are considered as an alternative for the glucose tracking due to their high sensitivity, superior stability, and fair selectivity. Metal oxides are one of the materials that has been recently studied in its glucose oxidation process for its sensing mechanism, operation pH restriction, and glucose oxidation selectivity.^5^ Whereas one of the bottlenecks for the metal oxide-based nonenzymatic glucose electrochemical sensor is the basic environment requirements (pH = 13.0), which limits the tendency of real application for the blood glucose monitoring considering the intrinsic property of neutral pH of blood.^6^ Therefore, investigating and creating a glucose sensor that can use metal oxide and tune its properties with certain doping materials such as platinum and gold is of great necessity in studying this research gap so that it can realize neutral pH glucose measurements. Prior studies^7−9^ have demonstrated that doping gold with iridium oxide has enabled and kept sensitivity in a basic environment but failed to address its sensitivity toward glucose oxidation process in neutral pH. Therefore, to further investigate how to tune the doping method in enabling glucose oxidation catalytic effect in neutral pH, this study focuses on building a Schottky barrier by contacting noble metal with metal oxides.

The Schottky barrier is the barrier formed when a p-type (its work function is larger than metal work function) or n-type (its work function is less than metal work function) semiconductor (metal oxide) is in contact with a noble metal, impeding electron transfer by forming a Schottky barrier height between the energy level of conduction band and Fermi level. The transfer of electrons is important in the direct electron transfer in nonenzymatic glucose oxidation process. The recently published papers have largely omitted such a Schottky barrier effect in tuning metal/metal oxides based sensing system. Therefore, it is worth further investigating the Schottky interface in enabling enhanced glucose sensing performance. The doping materials that are commonly used include metal, such as platinum, and gold^10−13^ etc. as each one of them can individually be applied for glucose detection. Whereas the doping of metal with metal oxides can create the Schottky barrier in the contact interface that reduces the barrier for the electron transfer, facilitating the glucose oxidation process on the metal oxide surface active sites.^14^ Therefore, it serves as a potential method for further tuning and adjusting the glucose oxidation parameters for higher sensitivity and better selectivity. In the meantime, metal oxide can serve as a pH responsive material for measuring the acidity and basicity for the local pH in the liquid environment, which can provide an alternative function and be constructed as a pH sensor *in situ* for accurately tracking and analyzing the pH in the process of glucose oxidation process, facilitating the glucose sensing mechanism study.

Whereas past research has briefly explained how the doping effects of noble metal to certain metal oxides play a role in adjusting the electron transfer barrier through the adjusted Schottky barrier.

The doping effects of metal oxides with noble metal has yet been fully investigated, though some researchers have briefly discussed about the potential mechanism.^14^ For example, Wang et al.^14^ have studied how the Schottky barrier has been increased when glucose molecules is in contact with the surface of the Au and nickel oxide and lose one electron to form gluconolactone so that it become positively charged. The positively charged electroactive intermediate can induce negative charges, resulting in increased Schottky barrier and further form reduced energy barrier so that accessing easier electron transfer, resulting in reduced energy for glucose oxidation process. However, this type of study has not been fully understood in other configurations in a wider and broader materials’ composition. A more systematic mechanism study of the metal/metal oxide is yet to be revealed. This research study has proposed using rhodium oxide as a metal oxide and doped with electrodeposited gold for the analysis of electron transfer in glucose oxidation process along with its pH sensing response, creating a dual sensor for revealing the sensing mechanism of glucose oxidation process with accurate pH monitoring. The dual functional sensor was realized by using either a multichannel electrochemical workstation or two electrochemical workstations in parallel for dual sensing of both pH and glucose molecules. The selectivity and sensitivity were further analyzed against a few common polysaccharides, enhancing the suitability of the constructed sensor for real application. The human serum sample was acquired from a local hospital, which was contributed from 20 diabetes patients. The sensing mechanism was thoroughly investigated in this research paper, and it can serve as a mechanism example for future glucose oxidation research in a neutral pH environment.

## Materials and Methods

### Reagents

Rhodium(III) chloride hydrate (RhCl_3_·xH_2_O) was obtained from Aldrich and used without further purification. Phosphate-buffered saline (PBS) and polyvinylpyrrolidone (PVP, M.W.=1,300,000) were purchased from Sigma-Aldrich. Uric acid (UA), L-(+)-ascorbic acid (AA) and D(+)-glucose were purchased from Thermo Fisher Scientific. Hydrogen tetrachloroaurate (III) trihydrate (HAuCl_4_·3H_2_O MW = 393.83 g/mol) was purchased from Alfa Aesar, United States. Fructose and lactose were obtained from Adamas. Galactose, Maltose, Sucrose, Xylose, and Sodium sulfate were purchased from Sigma.

Standard buffer solution (4.01 ± 0.02, 7.00 ± 0.02, 10.01 ± 0.02) were obtained from Hach LLC. Adjustable buffer solution was prepared by using 10 mM pH 7.4 phosphate buffer saline (PBS) which contained 137 mM NaCl, 2.7 mM KCl, 10 mM Na_2_HPO_4_, and 1.8 mM KH_2_PO_4_. All aqueous solutions were prepared with deionized water (18.2 MΩ·cm) generated by a Barnstead water system. Disodium hydrogen phosphate (Na_2_HPO_4_ F.W 141.96 g/mol) was purchased from SCR. Sodium chloride (NaCl, MW = 58.44 g/mol) was acquired from Titan. Monopotassium phosphate (KH_2_PO_4_), potassium chloride (KCl), sulfuric acid (H_2_SO_4_) and nitric acid (HNO_3_) were purchased from Tansoole and used as received without further purification. Phosphate-buffered saline (pH 7.2–7.4) was obtained from Biosharp.

### Electrospinning Synthesis of Rh_2_O_3_ NPs

According to the research of Dong et al.,^6^ first, 0.08 g of RhCl_3_·3H_2_O was dissolved in 4 mL of ethanol solution and added with 0.5 g of PVP. The mixed solution was under magnetic stirring overnight for a complete mixing. After that, the as-prepared viscous solution was electrospun using a 23-gauge needle with a flow rate of 0.2 mL/h at an applied voltage of 20 kV over an aluminum foil collector of 7 cm distance. The RhCl_3_/PVP nanofibers collected on the collector were then peeled off. After 4 h of the drying process at 95 °C in an oven, the as-prepared RhCl_3_/PVP precursor nanofibers were calcined under atmospheric pressure at 700 °C for 3 h with a ramp-up rate at 2 °C/min. The furnace was left to cool to room temperature naturally before collecting the as-prepared Rh_2_O_3_ nanoparticles (NPs) product.

### Preparation of Rh_2_O_3_ NPs Modified Glassy Carbon Electrode

To achieve the pure Rh_2_O_3_ modified glassy carbon electrode, the glassy carbon electrode (GCE, diameter of 3 mm) was polished with 3 μm, 1 μm, and 50 nm alumina slurries in sequence and then rinsed with deionized water. Then, the electrode was sonicated in ethanol and deionized water to remove residual alumina particles trapped at the surface and dried at room temperature naturally. The electrodes were ready for modification afterward. As for electrochemical pretreatment procedure, the precleaned electrodes were scanned for 10 cycles from −1.0 V to +1.0 V vs Ag/AgCl (Saturated KCl) in 2 M sulfuric acid at a scan rate of 100 mV/s until reproducible and smooth voltammograms were obtained. After that, the electrode was rinsed with deionized water for 1 min.

For GCE modification, following our prior research,^6^ 4–5 mg of Rh_2_O_3_ NPs were mixed with 1 mL of ethanol and sonicated for 40 min. Eight μL of Rh_2_O_3_ NPs suspension was dropped onto the surface of GCE. To trap Rh_2_O_3_ NPs on GCE, 2 μL of Nafion solution (1.0 wt % in ethanol) was further cast onto the top of Rh_2_O_3_ NPs. The as-prepared electrode is defined as Rh_2_O_3_ NPs/Nafion/GCE. The Nafion-coated glassy carbon electrode (Nafion/GCE) was also prepared as the control electrode for comparison in the following tests. Each as-prepared electrode was stored in deionized water to allow for a Nafion membrane for fully swelling.

### Synthesis of Nanocoral Gold

Based on the report of Sanzó et al.,^13^ nanocoral gold was directly prepared onto a single crystal solid (1, 1, 1) gold substrate by using chronoamperometric electrodeposition. The (1,1,1) gold (provided by Professor Li Yang’s group at XJTLU) was used as the working electrode and the platinum electrode and silver/silver chloride (Saturated KCl) electrode were used as the counter and reference electrodes. This three-electrode system was immersed in a solution of 0.01 M HAuCl_4_ and 2.5 M NH_4_Cl.^15^ Gold was then electrodeposited by applying a fixed potential of −3.0 V under stirring conditions for 20 s. The dark red electrodeposited Au with an area of 0.5 cm^2^ (1 cm × 0.5 cm square) can be obtained on a gold substrate for XRD, SEM, TEM and other characteriaation purpose. The nanocoral gold is coelectrodeposited with drop-casted rhodium oxide nanoparticles (Rh_2_O_3_ NPs) with aforementioned method to form nanocoral gold doped rhodium oxide electrode (GCE/Nanocoral Au/Rh_2_O_3_ NPs).

### Preparation of Buffer Solutions for pH Measurements

Different adjustable pH buffer was prepared by following the steps of Marzouk’s buffer preparation recipe^16^ in pH measurement. Briefly, pH 3.0–9.0 solutions were universal buffers containing 10 mM potassium hydrogen phthalate, 10 mM phosphate, and 10 mM Tris, while pH 10.0–13.0 solutions contained 50 mM sodium carbonate, 10 mM borax, and 140 mM of NaCl. Different pH of adjustable buffer was realized by adding different amounts of 1 M HNO_3_ or 1 M NaOH, and its pH was further confirmed by a commercial pH meter (Hach, USA).

### Preparation of Human Serum Sample by the First Affiliated Hospital at Soochow University

The human serum samples were collected by medical students and surgeons at First Affiliated Hospital at Soochow University from 20 randomly selected diabetes patients for fasting blood glucose measurements. The human serum was retrieved by centrifugation at 5000 rpm/min and stored at −20 °C before being shipped to the lab at Xi′an Jiaotong-Liverpool University. The human serum samples were completely melted and stabilized to room temperature before each patient’s fasting blood glucose level was tested. Then, these human serum sample’s glucose levels were tested by using a developed Rh_2_O_3_ NPs/Nafion/GCE to form a conventional three-electrode system in the presence of 0.1 M PBS buffer solution (pH = 7.2) for repetitive tests. A commercial glucose meter (Roche, Accu-Check Instant, CHN) was used to serve as a reference to compare it with electrochemical testing results. The related work has been granted with permission from the Ethic Committee of the First Affiliated Hospital of Soochow University with Grant No. 2024-Research No.025, and a permission from University Research Ethics Review Panel to conduct Research involving human participant Survey Respondent or Personal Data with Grant No. ER-SRR-11000018520231229134750 from Xi’an Jiaotong-Liverpool University (XJTLU) on January fifth, 2024. The informed consent forms were acknowledged and signed by participants and archieved at the hospital. The privacy rights of the participants were strictly followed by the requirements set by the ethics committees. The deidentification protocol was strictly followed per the research plan approved both at hospital and XJTLU.

### Apparatus and Electrochemical Measurements

A Raman spectrometer (Model Xplo RA, Horiba, France) was used to analyze the composition of Rh_2_O_3_ and Au thin film electrodeposited on the copper plate with a 514 nm wavelength laser. The electrodeposited thin-film materials were used for Raman Spectrometry characterization. The morphology of Rh_2_O_3_ and electrodeposited Au was observed by scanning electron microscopy (SEM, Model JSM 6510, JEOL, Japan), and energy dispersive X-ray spectroscopy (OXFORD Instruments) was employed for the analysis of element construction. Transmission electron microscopy (TEM, FEI, G2 F30) was applied for further morphological and crystalline structure investigation at working voltage of 300 kV. X-ray diffraction with Cu target at 45 kV and 40 mA (XRD model D8 ADVANCE, Bruker, USA) was used for the crystalline structure analysis. Cyclic voltammetry (CV) and Chronoamperometry were applied by using Electrochemical Workstation (Model CHI660E, CHI, China). A platinum column electrode (rod diameter 1 mm), and Ag/AgCl (saturated KCl) reference electrode were used for the three-electrode system pH measurement and glucose detection. Analytical balances (Model MS104TS, Mettler Toledo, Germany) were used to achieve the accurate weighing of reagents. Syringe Pump (Model 300, New Era Pump System, USA) was employed for injecting liquid into the syringes in electrospinning. The accurate pH value of different buffer solutions was tested by pH meter (Model Mettler Toledo AG 8603 Schwerzench, Switzerland). Heating Magnetic Stirrer (Model HS7 S25, IKA, Germany) was employed for the preparation of different reagents. Integrated muffle furnace (4-101P) obtained from Shanghai Huitai Equipment Manufacturing Co., Ltd. was used to heat the electrospun RhCl_3_ precursory nanofibers for oxidation process in transforming them into Rh_2_O_3_. Ultrasonic instrumentation (KQ-400DB, Shumei, China) was applied to disperse the nanocoral and nanoparticles in reaching a uniformly distributed suspension.

## Results and Discussion

### Morphology and Composition of Electrodeposited Rhodium Oxide Nanofibers and Gold Nanocorals

**Figure 1:**
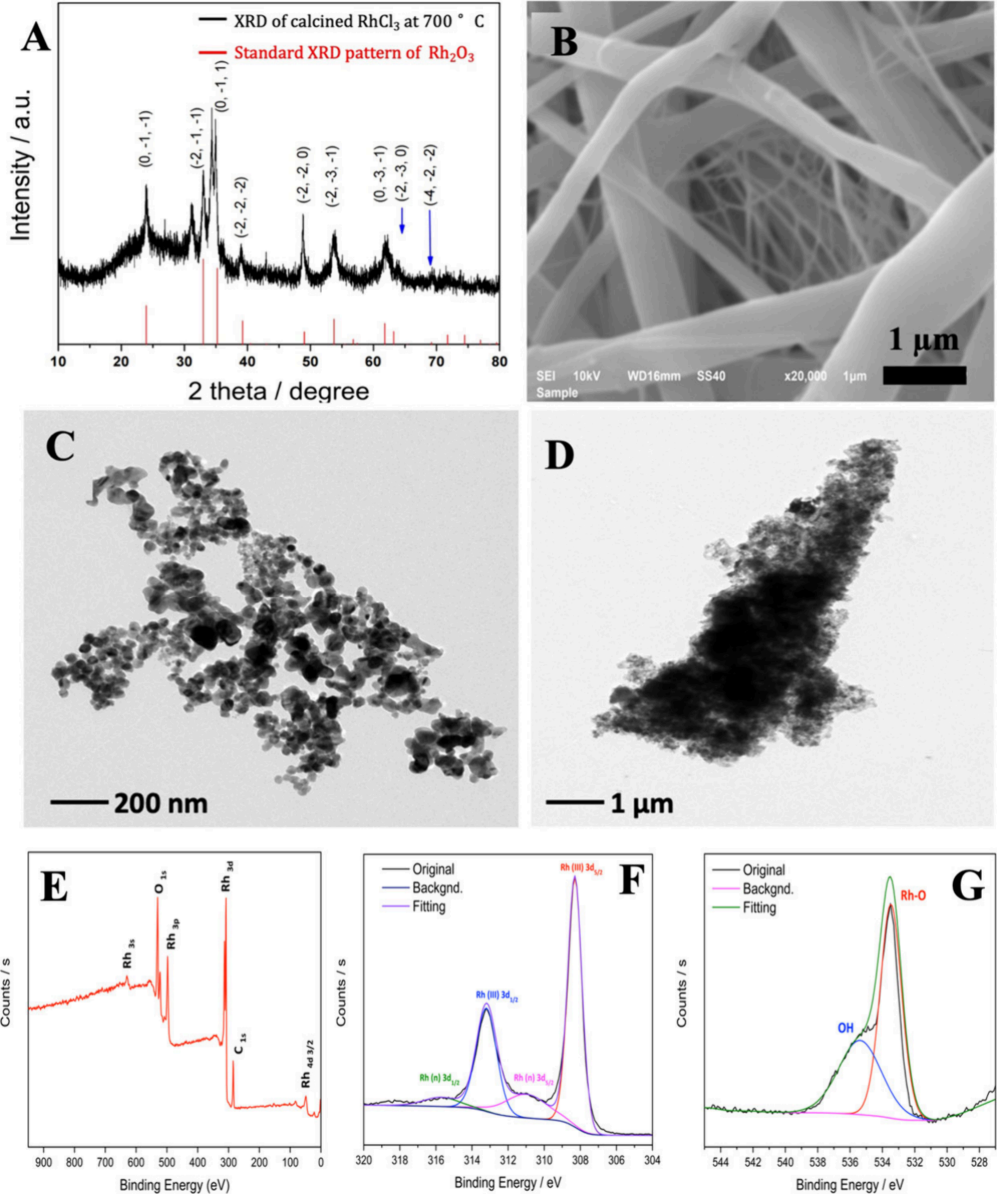
(A) XRD analysis of the annealed Rh_2_O_3_ nanocorals. The blue arrows indicate a minor shoulder peak with a different Miller index. (B) SEM image of precursory rhodium oxide microsize fibers. TEM images of Rh_2_O_3_ NPs (C) and Au (D). Color should be used to indicate the differences in print. The survey spectrum of XPS of Rh_2_O_3_ powder (E), Rh 3d spectra (F) and the O 1s spectra (G). Color should be used to indicate the differences in print.

The morphologies of the as-prepared RhCl_3_/PVP precursor and Rh_2_O_3_ powder are observed by SEM and presented in Figure 1B and Figure S1. According to Figure 1, the RhCl_3_/PVP samples can be defined as typical nanofibers with an average diameter of 288 nm. After calcination at 700 °C for 3 h, the surface changed from a fibrous structure into a nanocoral structure because of the degradation of PVP and decomposition of RhCl_3_ (shown in Figure S2).

The SEM images of electrodeposited gold on the (1,1,1) gold wafer was demonstrated at different magnifications shown in Figure S3. The morphology of the Au foam can be defined as porous and nanocoral foams which is similar to the Ag and Cu foams, which was reported with a pronounced 3D structure reported elsewhere.^13^

### XRD Analysis

For further analyzing compositional and crystal structure, XRD was used for characterization analysis. The XRD pattern (Figure 1A) of the calcined Rh_2_O_3_ NPs can be well-assigned to the characteristics peaks of the standard pattern of Rh_2_O_3_ from COD 1010584 database, which was revealed by peaks at 2θ values of 23.946°, 32.987°, 35.222°, 39.232°, 49.026°, 53.734°, 61.794°, 65.397, and 69.196°, corresponding to (0, −1, −1), (−2, −1, −1), (0, −1, 1), (−2, −2, −2), (−2, −2, 0), (−2, −3, −1), (0, −3, −1), (−2, −3, 0), and (−4, −2, −2) crystal planes,^17^ respectively. This result indicates the successful synthesis of rhodium oxide in well-separated 2θ peaks, matching well with a previous study.^6^Figure S4 shows the XRD pattern of the as-prepared microporous Au film and the achieved peaks matched the characteristics peaks (38.269°, 44.60°, 64.678°, 77.549° and 82.352°) of the face-centered cubic (FCC) Au crystal structure indicated by the orientations along the Au (1, 1, 1), (2, 0, 0), (2, 2, 0), (3, 1, 1) and (2, 2, 2) directions.^10^ According to Diaz et al.’ study,^18^ the XRD scanning results of the RhCl_3_ and PVP annealed at 800 °C for 1 to 2 weeks showed a peak at around 31°, which is ascribed as the Rh_2_O_3_ (0, 2, 0) crystal structure. In our study, the precursor was annealed at 700 °C for 3 h which led to the limited growth of this crystal plane. Therefore, the peak at 31° appears in the XRD pattern.

### TEM Analysis

Rh_2_O_3_ NPs and Au NCs were further characterized by TEM, as shown in Figure 1. The image in Figure 1C indicates the separated NPs of Rh_2_O_3_. The thin sample was frequently observed in TEM studies for Au nanobelts in Figure 1 (D).^19^ In addition, the SAED of Rh_2_O_3_ (Figure S5) and Au shows different *d*-spacing, which matches the XRD scanned results shown before and standard planes respectively (more details shown in Table S1 and Table S2). The TEM is in good agreement with XRD results in *d*-spacing. In this case, TEM results can further confirm the composition of electrodeposited Au and calcined Rh_2_O_3_ by corresponding to different crystal structures.

### EDX Analysis

To confirm the composition of prepared samples, the EDX spectrum (Figure S6B) demonstrated the presence of Cl, C and Rh, confirming the elemental existence of RhCl_3_ and PVP in precursor fiber-like structure. Figure S6A has demonstrated EDX spectrum for calcinated rhodium chloride along with PVP in precursor fibers, indicating a majority of Rh and O elemental existence in a 700 °C oxidation process besides carbon substrate for SEM EDX. This result is in line with other published data.^6^ After the precursor was calcined at 700 °C, the mapping can indicate that the RhCl_3_ was completely oxidized into Rh_2_O_3_ shown in Figure S6A. As for the electrodeposited Au shown in Figure S6C, the EDX scanning indicates the Au signal, which demonstrates the successful deposition of the gold layer on the surface of the electrode.

### XPS Analysis

After the existence of Rh and the O element was confirmed by EDX, the surface composition and the elementary chemical states of calcined Rh_2_O_3_ were further analyzed by XPS. Figure 1 (E) displays the XPS survey scan of calcined Rh_2_O_3_, from which the C, O, and Rh elements are distinctively detected. In this case, this result shows that RhCl_3_/PVP was completely oxidized into Rh_2_O_3_ after calcining at 700 °C for 5 h.

The Rh 3d core level spectrum shown in Figure 1 (F) demonstrated two contributions at 308.3 and 313.2 eV, which could be ascribed to Rh^3+^ 3d _5/2_ and Rh^3+^ 3d _3/2_, respectively.^20^ As for the doublets with Rh 3d_5/2_ and Rh 3d_3/2_ of approximately 311.0 and 315.7 eV also present in the Rh 3d spectrum, these high E_b_ values are typical for rhodium in a composition of complex organic materials, chloride or fluoride salts.^21^ Also, the rhodium hydroxide Rh(OH)_3_ might give impact in this area.^22^ Regarding the O 1s spectrum, the additional feature peaks at 533.5 and 535.4 eV show the oxides species of rhodium. As for the O 1s of Rh_2_O_3_ (Figure 1G), the specific peak was noted at 529.9 eV in previous study^23^ but the similar peak cannot be observed in our XPS analysis, which might be caused by the longer calcination duration (12 h) of RhCl_3_/PVP precursory nanofibers in this study. In addition, carbonaceous and adsorbed/capsulated H_2_O^24^ species were attributed to peak at 535.4 eV.

### Raman Spectrum

In Figure S7, Raman spectra of the calcined Rh_2_O_3_ NCs consist of four main predominant peaks at 268, 410, 555, and 593 cm^–1^, corresponding to E_g_ and A_1g_ modes of the orthorhombic Rh_2_O_3_ structure, which can confirm the existence of Rh_2_O_3_.^19^ Through characterizations discussed in sections 3.1 to 3.5, it can be concluded that the synthesis of rhodium oxide by precursor electrospun nanofibers has been confirmed and the doping of electrodeposited Au has been achieved together with rhodium oxide.

### Electrochemical Characterization of Nanocoral Au

The cyclic voltammetry (CV) scanning from −0.3 to 1.8 V vs Ag/AgCl (saturated KCl) in 0.1 M H_2_SO_4_ solution at a scan rate of 100 mV s^–1^ of three types of material for glucose sensing was shown in Figure S8. As for the electrodeposited Au, the peak occurred beyond 1.0 V and the catalytic current increases at the onset potential of around 1.1 V vs Ag/AgCl. According to Babar’s research,^25^ redox peaks of Au CV scanning can be considered as the redox reaction of the catalytic gold layer, which this peak cannot be obviously detected for bare gold wafer in H_2_SO_4_ solution. In addition, the electrodeposited gold modified with Rh_2_O_3_ NPs can detect a more specific redox peak and the oxidative peak not only occurred at around 1.05 V but also occurred a peak at 1.2 V as the oxidation reaction of Rh_2_O_3_ NPs.^26^ In this case, the real surface area of nanocoral gold can be measured by the area under the reductive peak in the polarization curve shown in Figure S8 and this area can be considered as the related charge value (*Q*) evaluated by dividing the integral (A V) by the scan rate (V/s). The value of an electroactive area can be calculated by the electrochemical active surface area equation (shown in eqns.1) where the electrochemical active surface area (ECSA) can be introduced and explained as in eq 1:
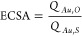
1Where *Q*_Au,O_ represents the charge associated with three different sensing materials, whereas *Q*_Au,S_ ascribes the theoretical charge calculated for an atomically 1 cm^2^ smooth Au electrode (386 μC/cm^2^).^27^

Based on this equation, the active surface areas of these three types of electrodes were determined by the minimum current observed at the decline of the redox peak (the results shown in Table 1).

**Table 1:** Electrochemistry Characterization Results of Three Types of Materials

Material	Geometrical area of the electrode/cm^2^	ECSA (electrochemical active surface area)/cm^2^	Roughness factor
Bare Gold wafer	1	28.50	28.50
Electrodeposited Au		77.72	77.72
Rh_2_O_3_ doped with electrodeposited Au		162.69	162.69

The roughness factor can be used for analyzing the oxidation of glucose for enhancing the faradic current based on the kinetically controlled sluggish reactions^13^ and roughness factor can be achieved as the ratio between the real surface area electrochemically measured and the geometrical area of electrode.^28^

The roughness factor is very important for the activity of the gold electrode, because the increase in surface roughness results in an increase in electrochemical activity.^29^ It is known that the roughness has an influence on kinetically controlled sluggish reactions such as oxidation of glucose for enhancing the faradic current. In contrast, the oxidation of ascorbic acid is a diffusion-controlled fast reaction, which is independent of the roughness of the electrode surface.^30^ It is evident that the roughness represents a very important aspect for the oxidation of glucose in biological fluids due to typical interfering components such as ascorbic acid and uric acid. The roughness factor (*R*_f_) was obtained by the ratio between the real surface area electrochemically measured and the geometrical area of the electrode and the result was shown in Table 1, which leads to the more active of sensing materials for glucose detection with the increase of *R*_f_.

### Rh_2_O_3_ NPs Modified Glassy Carbon Electrode for pH Sensing

To further explore the as-developed Rh_2_O_3_ NPs/Nafion/GCE application of a solid-state pH sensor, pH measurement was operated from pH 3 to 9 and then back to pH 3 as well as from pH 10 to 13 and then back to pH 10. Figure S9A,C shows the real-time EMF value with the changes of time and pH response of as-developed Rh_2_O_3_ NPs/Nafion/GCE and its Nernstian fitting are demonstrated in Figure S9B,D. Based on the Nernst eq (eq 2), the calculated Nernst constants of Rh_2_O_3_ NPs/Nafion/GCE are 28.1 mV/pH (between pH 3 and pH 9) and 24.4 mV/pH (between pH 10 and 13), respectively. To ensure the role of Rh_2_O_3_ during pH monitoring, the Nafion/GCE was used for the same pH test as the controlled experiment in Figure S10 and the pH response is unstable in a basic environment and is low in an acid environment. In conclusion, as-prepared Rh_2_O_3_ NPs have good performance (high sensitivity, stability, and fast response) in the pH range (3 to 9 and 10 to 12) and Nafion/GCE shows poor or almost no pH response. eq 2 demonstrates the Nernst equation in calculating the theoretical Nernstian constant.
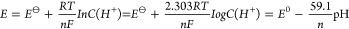
2at 25 °C

Additionally, the three- and two-electrode system was also studied for these kinds of materials. It is admitted that the two-electrode system is a conventional system for open circuit potential-time (OCPT) testing while the added counter electrode into this method can be explained by the Randles circuit for its enhanced Nernst constant. This is because the redox reaction including the attachment of proton and the acceptance of electron or the release of hydroxide ion and electron on the rhodium oxide surface^6^ (shown in eq 3, eq 4, eq 5, and eq 6) may cause current flow so that the existence of IR drop can offer a more pronounced and responsive (larger) Nernst constant in the three-electrode system in potentiometric pH measurement (the compared results shown in Figure S11). Figure S11A, S11B, and S11C compares the different responses of three-electrode and two-electrode systems in the range of pH 4 to 10 with three standard pH buffers.
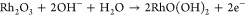
3
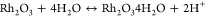
4
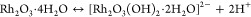
5
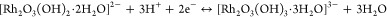
6

**Figure 2:**
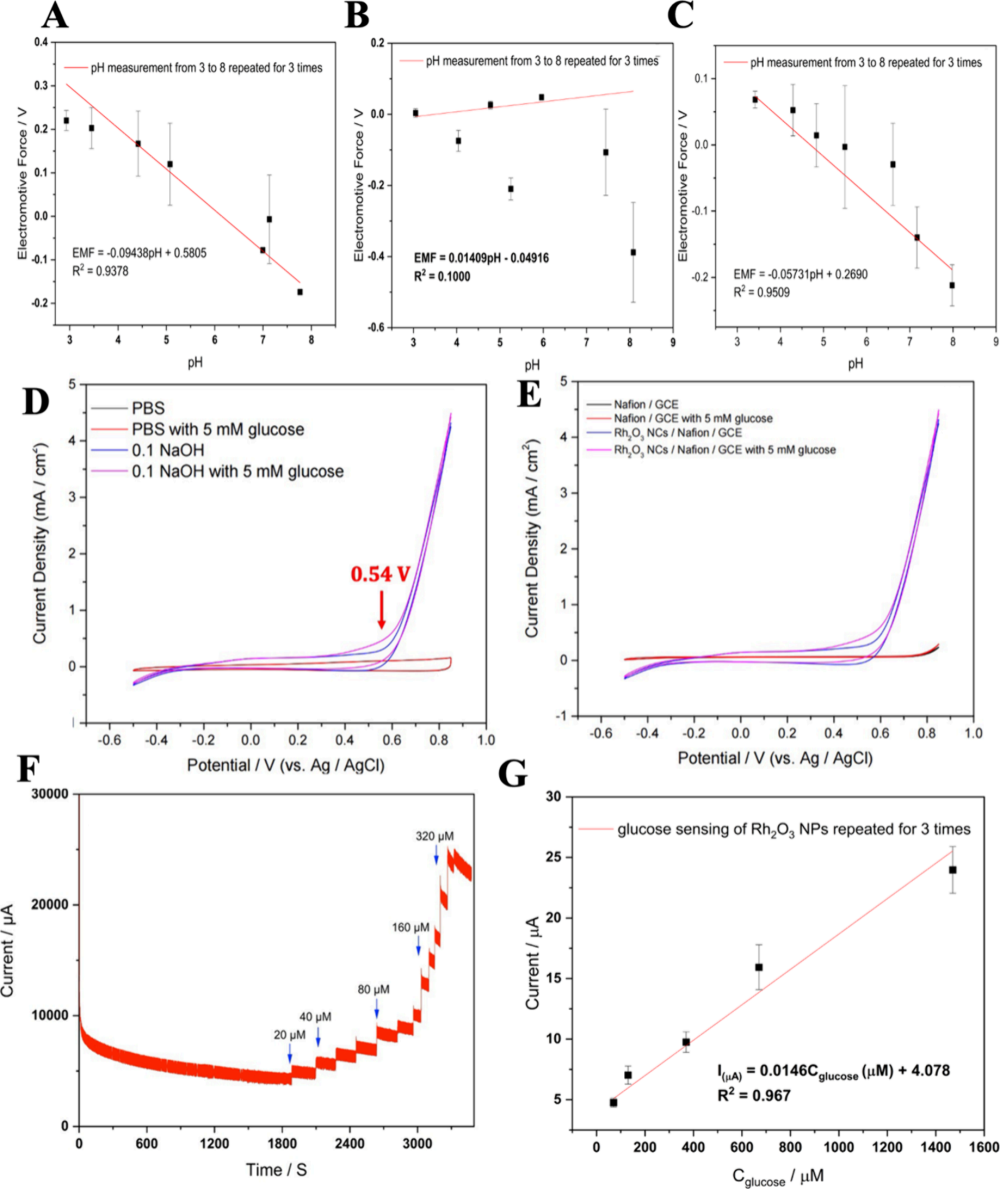
Fitted equation (slope indicates Nernst constant) between electromotive force (EMF) and time for the (111) Au (A), nanocoral Au on (111) Au wafer (B) and Rh_2_O_3_ NPs dropped on nanocoral Au (C) during reversible pH titration cycles in the range of pH 3.0–8.0. Color should be used to indicate the differences in print for A and B. The CVs of Rh_2_O_3_ NPs/Nafion/GCE in 10 mM phosphate buffer saline (pH = 7.4) and with an absence and presence of 5 mM glucose in 0.1 M NaOH electrolyte, respectively. (D). The CVs of the Rh_2_O_3_ NPs/Nafion/GCE and Nafion/GCE in 5 mM glucose in 0.1 M NaOH electrolyte, respectively (E). Amperometric response of the Rh_2_O_3_ NPs/Nafion/GCE to successive addition of glucose at an applied potential of +0.54 V (vs Ag/AgCl). (F) and the corresponding raw data (dots) and the linear fitting curve (red solid line) (G). Color should be used to indicate the differences in print for A and B.

In this case, the mixed material (Rh_2_O_3_ dropped on nanocoral Au) was therefore also used for the pH sensing three-electrode system for solid-state pH measurements application. According to Figure 2C, the Nernst constant of this mixed pH sensor is around −65.42 mV/pH in the range of 3 to 9. In addition, the nanocoral Au sensor and (111) Au were also prepared for the same pH measurement to serve as a control experiment in confirming the main active material of pH sensing, which is shown in Figure 2A and 2B. Compared to the pH response of nanocoral Au sensor and (111) Au, these two kinds of materials showed low stability during the pH test. In this case, although the Au materials can show a response with the changes of pH values, the pH response is lower and less stable than the combined Rh_2_O_3_ NPs/Nafion/GCE pH sensor, which can confirm the main materials role of Rh_2_O_3_ for pH sensing. In conclusion, the Rh_2_O_3_ NPs can not only improve the sensitivity of glucose sensing in a neutral environment but also offer pH responsive performance from pH 3 to 12 (as shown in Figure S9).

### Rh_2_O_3_ NPs Modified Glassy Carbon Electrode for Nonenzymatic Glucose Detection in Basic Environment

To obtain the applicability of Rh_2_O_3_ NPs for glucose oxidation in a basic environment, the cyclic voltammetry (CV) of the Rh_2_O_3_ NPs/Nafion/GCE in neutral PBS buffer (Figure 2D) and 0.1 M NaOH (Figure 2A) were investigated in the absence and presence of 5 mM glucose ranging from −0.5 V to +0.85 V vs Ag/AgCl (saturated KCl) at a scan rate of 100 mV/s. Based on the prior literature,^6^ the mechanism of metal oxide sensing glucose oxidation in alkaline environment is considered as the reaction between metal oxide and OH^–^ ions and the release of electrons and generation of metal oxide hydroxide. In this case, the Rh_2_O_3_ glucose sensing mechanism can be defined as follows in the eq 7 and eq 8 processes:
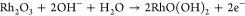
7
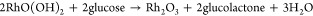
8

According to Figure 2A, no glucose oxidation peak can be observed on the Rh_2_O_3_ NPs/Nafion/GCE electrode in PBS buffer solutions (pH = 7.4), while evident peak can be observed at 0.54 V, indicating the requirement of hydroxyl ion in glucose oxidation on Rh_2_O_3_ NPs in basic environment. The voltage at 0.54 V (vs Ag/AgCl) was also further used for the amperometric study in the next section. In addition, the controlled experiment was designed by using Nafion/GCE for the similar glucose detection in PBS buffer and 0.1 M NaOH shown in Figure 2E, validating the role of Nafion not interfering with the glucose oxidation process. There are no significant peaks with the presence or absence of 5 mM glucose in a neutral or basic environment for Nafion/GCE electrode, which means that the Nafion/GCE shows a poor glucose sensitivity and the Rh_2_O_3_ NPs are responsible for glucose detection.

As demonstrated in Figure 2A, the amperometric study has applied 0.54 V (vs Ag/AgCl) as applied potential for further study with modified Rh_2_O_3_ NPs/Nafion/GCE electrode. Figure 2F shows typical amperometric responses of the prepared sensor to successive addition of glucose in 0.1 M NaOH electrolyte with well-defined steps in an interval of 180 s. The corresponding calibration line (current vs glucose concentration) was shown in Figure 2G with a calculated *R*^2^ = 0.993. With the increase of glucose concentration, the amperometric current grows proportionally and demonstrates a concentration-dependent behavior with good linearity. The limit of detection (S/N = 3) was calculated to be 60 μM, while the sensitivity of the modified Rh_2_O_3_ NPs/Nafion/GCE was 20.17 μA mM^–1^ cm^–2^ under basic condition. This number was in well agreement with the past publication^6^ for using rhodium oxide serving as nonenzymatic glucose sensor.

### Improvement of Rh_2_O_3_ NPs Doped with Electrodeposited Gold for Nonenzymatic Glucose Detection in Neutral Environment

**Figure 3:**
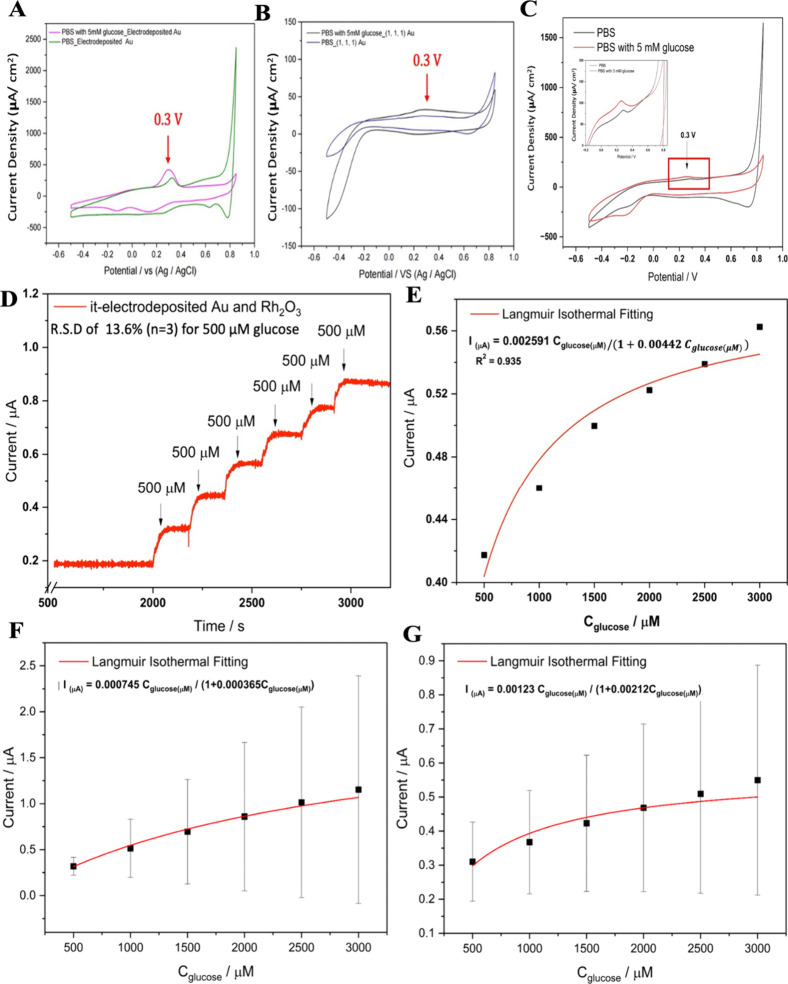
CVs of electrodeposited Au (A), bare (111) Au (B) and doped Rh_2_O_3_ on the electrodeposited Au wafer (C) in 10 mM phosphate buffer saline (pH = 7.4) with the absence and presence of 5 mM glucose. Color should be used to indicate the differences in print. Amperometric response of Rh_2_O_3_ NPs doped on electrodeposited Au wafer to successive addition of glucose at an applied potential of +0.30 V (vs Ag/AgCl) (D), and the corresponding raw data (dots) and the Langmuir isothermal fitting curve of Rh_2_O_3_ NPs doped on electrodeposited Au wafer (red solid line) (E). The intrastability test of electrodeposited Au doped with Rh_2_O_3_ NPs for glucose response tests (F) and the interstability test of electrodeposited Au doped with Rh_2_O_3_ NPs for glucose response tests (G).

Based on the above glucose sensing results, it is admitted that Rh_2_O_3_ NPs shows good sensitivity of glucose measurement at high pH values using the amperometric technique but is not responsive for the oxidation of glucose in PBS neutral solutions because a high amount of chloride ions is adsorbed onto the surface in neutral conditions.^29^ In this case, as for the Rh_2_O_3_ NPs/Nafion/GCE glucose test, the samples have to be adjusted for its pH values before the detection while the pretreatment of samples can introduce variations for uncalibrated samples.^13^ In order to solve the inhabitation of glucose oxidation caused by chloride ions, a porous nanostructure is able to avoid the interference during the electrooxidation.^30^ In this study, the electrodeposited nanocoral Au was selected for improved glucose sensing in neutral condition. Figure 3A shows the scan of nanocoral Au in PBS solution in the absence and presence of 5 mM glucose. There are two broad peaks that can be assigned at around 0.280 V (reduction) and 0.320 V (oxidation), which represents the OH^–^ desorption and adsorption from the Au surface.^31^

**Scheme 1:**
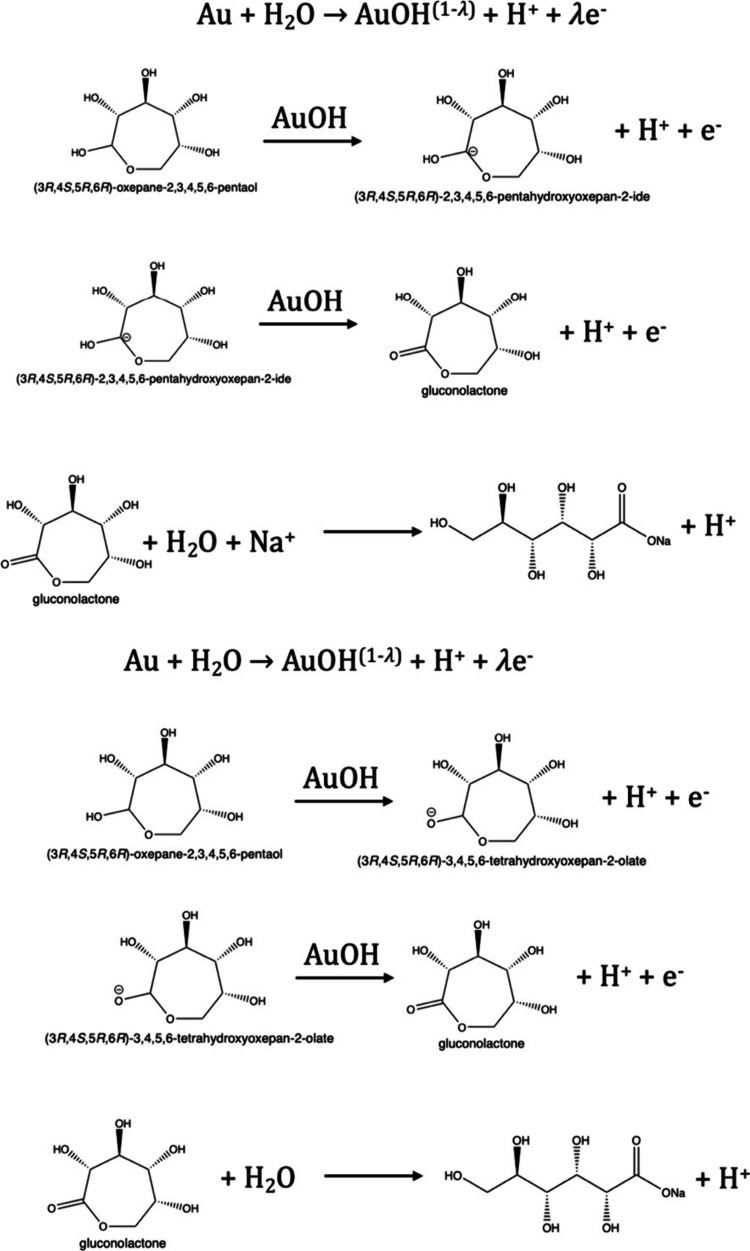
Glucose Oxidation Involved with Gold Participation Mechanism Demonstration The mechanism was redrawn by referring to the work presented by Hsiao, M.W.^31^ Reproduced with permission from Ref,^31^ Copyright 1996, Journal of the Electrochemical Society.

As for the CVs result of electrodeposited Au after adding 5 mM glucose, the anodic current increases dramatically from 0.2 to 0.4 V, and a sharper peak can be detected at 0.3 V, marked as the glucose oxidation (as shown in Figure 3B). In addition, Wang et al. reported two broad peaks at about 0.15 and 0.30 V which are defined as the oxidation process of glucose to gluconolactone.^32^ According to the prior report,^31^ there are two main types of the glucose sensing for Au: the first step is the transform of Au into AuOH species on the surface and the oxidation of glucose can exist due to the AuOH groups and hemiacetal groups, which is distinguished by the leaving group, hydrogen ion from hydroxyl groups or alkyl groups. After which, as formed gluconolactone is hydrolyzed to produce gluconic acid (more details are shown in Scheme 1).

For comparison, the CV curves of a bare (1, 1, 1) Au on glass are displayed in Figure 3B, and there is a weak peak at around 0.3 V, presenting weaker current responses than those of the electrodeposited nanocoral electrode. As the electrodeposited nanocoral Au can detect 5 mM glucose in PBS solution, the applied potential (+ 0.3 V) is applied for amperometric measurement as shown in Figure 3D. In this case, typical amperometric responses of the as-electrodeposited Au wafer to successive addition of glucose in PBS solution can be obtained and shown in Figure S12A. However, the corresponding calibration line (current vs glucose concentration) shown in Figure S12B indicates that the glucose sensitivity of electrodeposited nanocoral Au (limit of detection = 450 μM) is lower in neutral environment than that of the Rh_2_O_3_ NPs in basic environment. (glucose sensitivity is 0.46 μA mM^–1^ cm^–2^, which is close to the value 0.5 μA mM^–1^ cm^–2^ reported in previous work^13^).

Therefore, the Rh_2_O_3_ NPs doped with the electrodeposited nanocoral Au wafer is considered as the alternative pathway for more sensitive glucose detection in a neutral environment. According to Figure 3C, it also shows the specific peak at around 0.30 V, which is defined as the transfer of glucose into gluconolactone. In this case, 0.3 V voltage was also chosen as the applied voltage for the amperometric measurement of rhodium oxide doped nanocoral gold. As for the detailed amperometric response, it is notable that the glucose detection of Rh_2_O_3_ NPs doped on an electrodeposited nanocoral Au wafer (shown in Figure 3E) has a wider sensing range compared to the glucose sensitivity of the electrodeposited nanocoral Au wafer (glucose sensitivity is 3.52 μA mM^–1^ cm^–2^). Each electrode was used for glucose sensing for 3 times and the relative standard deviation (R.S.D) is 4.19%. (The other three amperometric response test results were shown in Figures S13, S14 and S15 against a gradient of concentrations of glucose molecules). Additionally, five electrodes fabricated by the same method was prepared for the interstability test and the R.SD is 13.6%, which indicated the high reproducibility of this kind of technique. As the electrochemical oxidation of glucose on Rh_2_O_3_ NPs is considered as a surface catalytic reaction, Langmuir isothermal equation can be adapted for data processing based on Langmuir isothermal theory.^33^ The corresponding fitting curve is represented by eq 9 as follow:
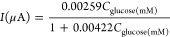
9After one month storage of this kind of electrode at room temperature exposed in the air environment, the stability test of glucose was investigated, and the results are shown in Figure S16. The electrode current response to the same concentration of glucose has dropped to one-fifth of the original current of the prepared electrode. This demonstrates the prepared sensor has a poor stability in one-month storage period. The future research should be followed up in further improving the long-term stability. The possible reason for the decayed response is the electrode fouling and Schottky interface disassembly^34^

**Scheme 2:**
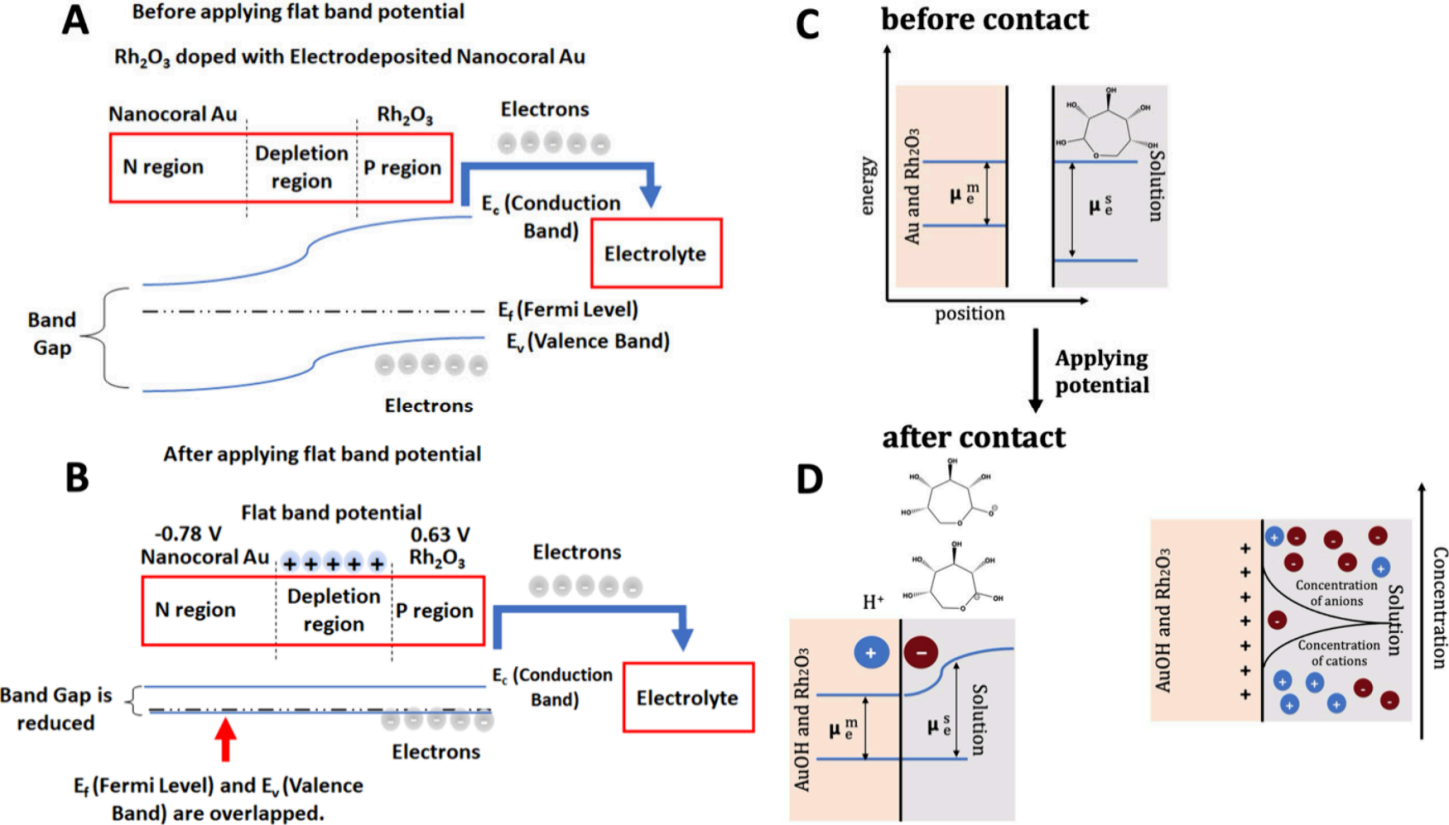
Application before Flat Band Potential and the Description of Crossed Fermi Level with Valence Band in Forming Metallic Conductor after the Applied Flat Band Potential Color should be used to indicate the differences in print. Electrochemical equilibrium at an electrode-electrolyte interface after applying 0.3 V during the glucose determination of electrodeposited Au and Rh_2_O_3_ NPs electrode. μ stands for the chemical potential. μ_*e*_^*m*^ stands for chemical potential on the metal oxide/metal electrode. μ_*e*_^*s*^ stands for the chemical potential in the solution.

For further study on the mechanism of improved materials for glucose sensing, the flat band potentials of nanocoral Au and Rh_2_O_3_ doped with the nanocoral Au were investigated by Mott–Schottky plots (Figure S17). Flat band potential is a special circumstance when the conduction band and valence band meet, and the band bending effect is no longer taking the dominance in the case of Schottky barrier formation. With the flat band potential application, the conduction between the metal oxide and metal (heterojunction interface) has greatly enhanced and it behaves as a metallic material, enabling the electron transfer and accessing lower onset potential for oxidation reactions. It can be noted that the Rh_2_O_3_ NPs and electrodeposited Au are considered as a p-type and n-type semiconductor according to the negative and positive slope in Figure S17A and B, respectively,^35^ while the Mott–Schottky plots of Rh_2_O_3_ NPs@nanocoral Au (V-shape) can be confirmed as the n-type and p-type mixed material due to the p–n junction was framed.^36^Figure S17C is a good example of the p–n junction formation due to the contraction of p-type material (Rh_2_O_3_ NPs) and n-type material (nanocoral Au). Generally, the flat-band potential, defined as the potential at which the Fermi level of the semiconductor is flat (conduction band and valence band meet) and there is no charge depletion region. It can be measured that for the flat-band potential of Rh_2_O_3_ and nanocoral Au could be identified as 0.63 eV and −0.78 eV, respectively in Figure S17A and S17 B*x*-axis intercept, which confirms the applicable main voltammetric peak in Figure 3C (∼0.3 V vs Ag/AgCl (saturated KCl)). This conclusion can be explained by the semiconductor theory, the capacitance of the space-charge region of a p-type semiconductor shown in eq 10:
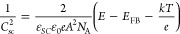
10where ε is the relative permittivity (dielectric constant), ε_0_ refers to the vacuum permittivity, e represents the charge of the electron, *N*_A_ is the acceptor concentration determined by the slope of the linear relation, *E* is the applied potential and *E*_FB_ is the flat band potential. According to this equation, measuring the interception of the linear region with the axis standing for the applied potential can yield the flat-band potential. In this case, this relationship indicates the changes of the space-charge area when depleted and an external polarization potential *E* is applied for further detection (ϕ_SC_ = *E* – *E*_FB_).^37^ Based on these references,^38^ the Fermi level can be explained specifically by the microcosmic construction of electrode and electrolyte and Alberto et al.^39^ reported the artificial changes of Fermi level for semiconductor by applying external voltages and built up the relationship between Fermi level and flat band under specific external potential. In this case, the position of the semiconductor is lower than that of the electrolyte, and the electrons flow from semiconductor to electrolyte, which causes an accumulation layer of vacancies (holes) in the valence band due to the upward bent bands. The accumulation of these vacancies at the surface of materials may cause a positively charged region, and the Fermi level can be adjusted into a lower position. If the Fermi level is very close to the border of the valence band, which is easily crossed, the charge accumulation occurs, and the metallic conductor appears. Scheme 2A and B illustrates such an artificial Fermi level cross with valence band process. Therefore, the electrons from the semiconductor move to the electrolyte interface leading to the oxidation of metal (electron leaving from semiconductor-metal interface). This is the reason why only applying this potential range (−0.5 V to 0.85 V) above the flat band potential can cause the transition of electrons between electrodes (Rh_2_O_3_ and nanocoral Au) and electrolytes for glucose sensing. However, some species (AP, ascorbic acid, uric acid) are likely to be oxidized at 0.7 V of applied potential, which leads to low selectivity during glucose determination of human blood. In this case, the operating potential cannot be set over 0.7 V.^40^ Additionally, glucose oxidation occurs at the applied potential of 0.3 V (as shown in Figure 3C) so the applied potential set at 0.3 V can adjust the Fermi level of Au so that the electrons can transit from the valence band to the conduction band (the first step shown in Scheme 1) but can cause the next step of glucose sensing on the AuOH surface as well. As for the glucose oxidation on the AuOH surface, this reaction can be explained by the electrochemical double-layer^41^ which is the electrochemical equilibrium at an electrode–electrolyte interface shown in Scheme 2 C and Scheme 2D. Before applying the potential at 0.3 V, there is no contact between the solution and the surface of Au due to the difference in electrochemical potential of the electrons in the Au and Rh_2_O_3_ electrode and the glucose. After the gold oxidation at a suitable operating voltage based on the Mott–Schottky test, the Fermi level of metal/metal oxide can be adjusted at the lower level for the electron transition so that the AuOH and H^+^ occur, which leads to the balance between positive charges on the electrode surface (AuOH) and the negative ions in the solution. In this case, amperometric response enhances with the addition of glucose because the ionic charge in the solution equals the charge on the sensing electrode (overall charge neutrality).

Table S3 has summarized and compared the constructed rhodium oxide nanoparticle glucose sensor performance in basic electrolyte, neutral electrolyte, and rhodium oxide doped with noble metal gold for its sensing performance in limit of detection, sensitivity, linear range, and sensing pH condition.

### Selectivity of Rh_2_O_3_ NPs Doped on Electrodeposited Nanocoral Au Wafer for Nonenzymatic Glucose Detection in Neutral Environment

As for the real test samples, the concentrations of 4-acetoamenophen (4-AP) ascorbic acid (AA) and uric acid (UA) are preapred depending on the specific concentrations of these interferences in human’s blood.^6^ As for the selectivity test, other sugar including fructose, galactose, lactose, maltose, sucrose and xylose were prepared with concentrations of about one-tenth of that of the glucose^10^ in human serum sample. Three types of molecules, including UA, AA, and 4-AP may affect the precise results of electrochemical glucose sensing according to our prior experience. In this case, Rh_2_O_3_ NPs doped on electrodeposited Au wafer was investigated for the selectivity test of AA and UA. In this study, the glucose concentration is prepared for 500 mM, which is high enough to study the effects of other interferences to the glucose response in normal physiological level^42^ and the concentration of 4-AP, AA and UA were prepared at 10 μM, 200 μM and 40 μM, respectively. These concentrations were prepared considering the solubilities of the molecules in the electrolyte buffer. According to Figure S19, the result demonstrates that three-dimensional porous Au and Rh_2_O_3_ electrode could offer a much higher response to AA and UA, especially an evident increase of addition of AA, and this pronounced increasing can be distinguished by the abrupt response to differentiate its response with glucose molecules. As for the peak caused by subsequent addition of UA, the current dropped into the baseline level the same as the prior 500 μM glucose response in 1 min duration. In addition, a strong response was shown with the additions of glucose while the current dropped quickly when adding other saccharides and finally returned to its baseline in 10 s (shown in Figure S19). Therefore, this selectivity test demonstrates good selectivity of the developed nonenzymatic glucose sensor. The response of glucose to other saccharides behaved differently, so that a selective detection can be realized.

Table 2 summarizes the past enzymatic and nonenzymatic glucose sensors for different sensing materials along with its sensing performnace. The sensitivity, linear range, limit of detection and sensing environment are also emphasized to differentiate the advantages of the proposed sensor in neutral pH sensing capacity with metal oxide materials.

**Table 2:** Enzymatic and Nonenzymatic Glucose Sensor Sensing Performance Comparison Table

Sensing Mechanism	Sensing materials	Sensitivity (μA mM^–1^ cm^–2^)	Linear range (mM)	Detection condition	Limit of Detection	Advantages	ref
Enzymatic	Glucose oxidase/polyindole/MWCNT/Screen-printed carbon electrode	182.9	0.01–100 mM	PBS buffer (pH 7.4)	0.01 mM	Good selectivity	(43)
	Glucose oxidase/bilirubin oxidase (BOD)/highly porous gold(hPG)	14.13	50 μM–1 mM	0.1 M pH 7.4	50 μM	Accompanied with self-powered fuel cells	(44)
Nonenzymatic	Au/Co_3_O_4_ hybrid	12500	0.001–10	0.5 M KOH	5 nM	High selectivity against UA, AA, AP, etc.	(45)
	CuO nanotubes-graphene	1360	0.002–4	0.1 M NaOH	0.7 μM	Good selectivity against normal electroactive compounds	(46)
	ZnO nanorods	5.601	0.001–0.01	0.1 M PBS	0.5 μM	Not mentioned	(47)
	Coral-like PtAu-MnO_2_	58.54	0.1–30	0.1 M PBS	0.02 mM	Good selectivity against AA, UA and DA	(48)
	Rhodium Oxide Nanocoral	11.46	Nonlinear fitting up to 6 mM	0.1 M NaOH	3.1 μM	Advantages in wide linear range	(6)
	Rhodium oxide doped Au	3.52	Up to 3 mM	0.1 M PBS pH 7.4	20 μM	Adjusted pH sensing range from basic to neutral	This work

### Specific Application in Diabetic Human Serum

**Figure 4:**
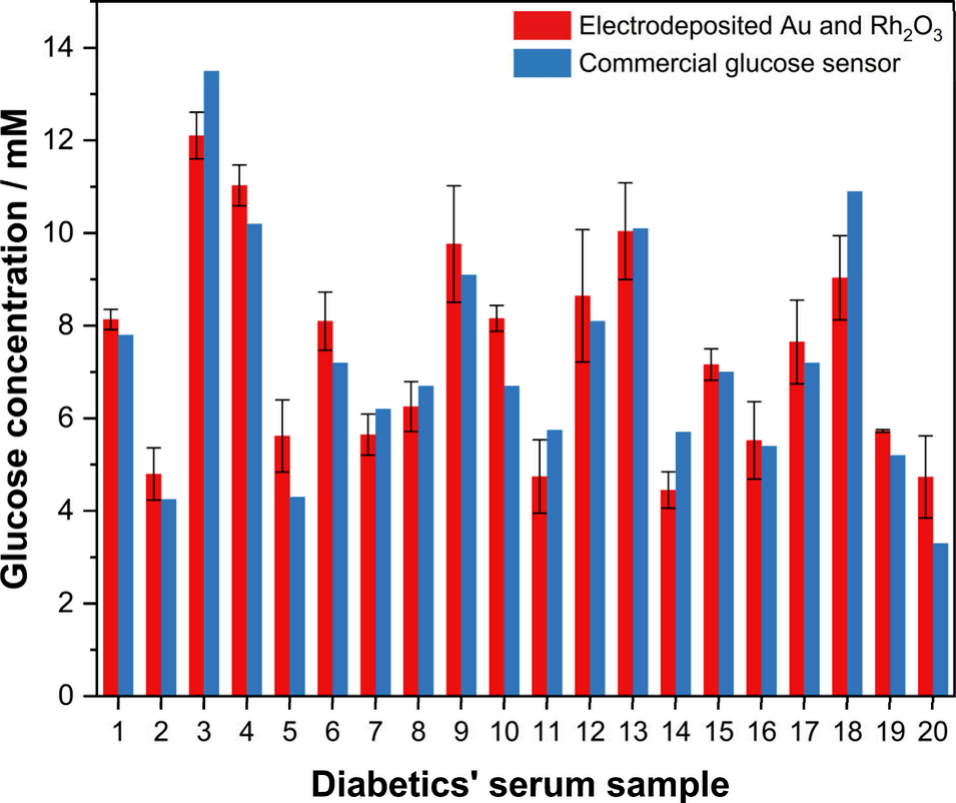
Glucose concentration in human serum sample was determined by a commercial glucose meter (Roche, Accu-Check Instant, CHN) and the prepared sensor in this study. Error bars indicate the standard deviation for multiple measurements (*n* = 2 for commercial glucose meter and *n* = 3 for the as-developed sensor). Color should be used to indicate the differences in print.

The determination of glucose in serum was necessary for the specific employment of the prepared glucose sensor, so 20 diabetic fasting blood serum samples were collected from the pathology laboratory of the First Affiliated Hospital of Soochow University. According to Figure S18A, the concentration of glucose in 450 μL of serum sample can show the obvious amperometric response during the first sequential injection in 10 mM phosphate buffer saline (pH = 7.4). In this case, 100 μL injection of the test sample was used for determination of the unknown concentration. The characteristic response upon the successive addition of human serum samples with glucose concentration was shown in Figure S18A. As the linear relationship between current and concentration of glucose can be achieved by the same injection of standard 100 mM glucose solution in 10 mM phosphate buffer saline (pH = 7.4) in Figure S18B and S18C, the corresponding concentration of each sample can be determined based on this equation (*I* (μA) = 0.00137 C_glucose_ + 0.353, *R*^2^ = 0.997, shown in Figure S18C). Meanwhile, the glucose concentrations of these 20 human serum samples were determined by a commercial glucose meter (Roche Instant Accu-Check) for comparison (shown in Figure 4 below). According to Table S4, only one absolute value of the *t* test exceeded the critical value *t*_2_ (9.92 at 99% of significant level), which indicates an overall good agreement between the glucose concentration of serum tested by commercial glucose meter and prepared sensor in this study. Therefore, the Rh_2_O_3_ doped electrodeposited Au sensor can be employed for specific glucose sensing in human serum with precise and repeatable values.

## Conclusion

The proposed rhodium oxide doped electrodeposited Au (Rh_2_O_3_@nanocoral Au) has been demonstrated with superior electrochemical active surface area and roughness factor, and it has great enhancement to the glucose oxidation compared to the rhodium oxide nanoparticles (Rh_2_O_3_ NPs/Nafion/GCE) based sensor for neutral pH detection. The rhodium oxide nanoparticle sensor has demonstrated the detection of a limit of 450 μM and sensitivity of 0.46 μA mM^–1^ cm^–2^. While the rhodium oxide doped electrodeposited gold has shown better performance with improved sensitivity of 3.52 μA mM^–1^ cm^–2^.

The potential mechanism for the rhodium oxide and electrodeposited Au has been investigated by Mott–Schottky analysis, and it has illustrated how the electrons were transferred to electrolyte from metal oxide and how the Fermi level can be adjusted to a lower position for easier electron transfer. This research has novelty explained how the doping of gold with rhodium oxide facilitated the electrons transfer in the glucose oxidation process, leading to a possible path in illustrating other types of metal oxide based noble metal systems.

It provides an alternative mechanism illustration for the glucose oxidation study for nonenzymatic system. In addition, the proposed system can serve as a pH sensor that can fill in the pH sensitivity property while keeping the glucose sensitivity intact.

This constructed dual sensor has great potential in advancing selective glucose oxidation in the next generation of glucose determination for nonenzymatic glucose based continuous blood glucose monitoring through multichannel electrochemical workstation or two ones in parallel. This work has laid out a solid foundation for the mechanism study of glucose oxidation.

## Supporting Information Available

Additional experimental details, methods and materials, tables, including characterization and pH sensing performance and human serum glucose tests (PDF)
