# Supplementary material for: Schottky Interface Enabled Electrospun Rhodium Oxide Doped Gold for Both pH Sensing and Glucose Measurements in Neutral Buffer and Human Serum
Source: Langmuir. 2024 Sep 17;40(39):20797–810. doi: 10.1021/acs.langmuir.4c02999 (PMC11447893; doi:10.1021/acs.langmuir.4c02999)
Supplement: Supplementary file 1 — la4c02999_si_001.pdf [file la4c02999_si_001.pdf]

Electronic Supporting information

Schottky Interface Enhabled Electrospun Rhodium Oxide Doped Gold for both pH Sensing and Glucose Measurements in Neutral Buffer and Human Serum

Weiyu Xiao[1], Mingman Li[1], Danlei Li[1], Bo Shi[2], Runze Zhong[2], Yiyuan Zhao[2], Qingliang Tai[2], Songbing He[2*], Qiuchen Dong[1,*]

Affiliation:[1]: Department of Chemistry, School of Science, Xi'an Jiaotong-Liverpool University No. 111 Ren'ai Road, Suzhou Industrial Park, Dushu Lake Higher Education and Innovation Park, Postcode: 215123, Jiangsu Province, People's Republic of China

[2]Department of General Surgery, The First Affiliated Hospital of Soochow University, No. 188 Shizi Street, Suzhou 215006, Jiangsu Province, People's Republic of China

[*]Corresponding Author

ORCID: Weiyu Xiao: https://orcid.org/0009-0005-3484-9832

ORCID: Qiuchen Dong: https://orcid.org/0000-0003-4100-3921

Email: hesongbing1979@suda.edu.cn; qiuchen.dong@xjtlu.edu.cn;

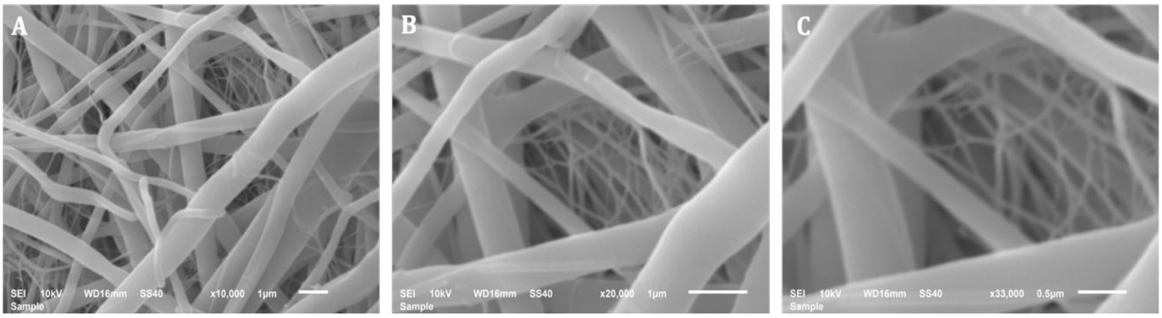

Figure S1. The morphology of precursor dried for 48 hrs. at different scale bars – 1 μm (A) and (B) and 0.5 μm (C) under SEM.

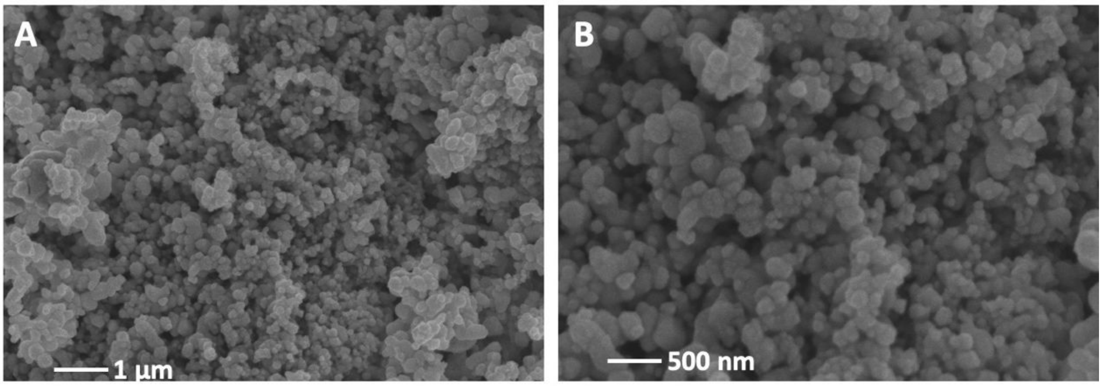

Figure S2. The morphology of electrospun $RhCl_3$/PVP precursory nanofibers calcinated at 700 °C for 3 h, which it was taken under 10 KV of a scanning electron microscopy. The scale bar of each figure is 1 μm(A), and 500 nm (B).

Figure S3. The morphology of as-electrodeposited Au nanocorals. The scale bar is labeled with 1

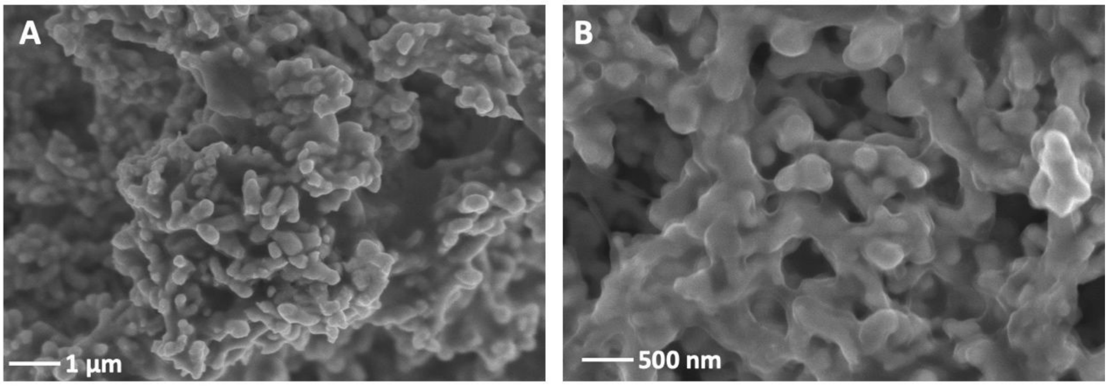

μm(A), 500 nm (B) in each figure.

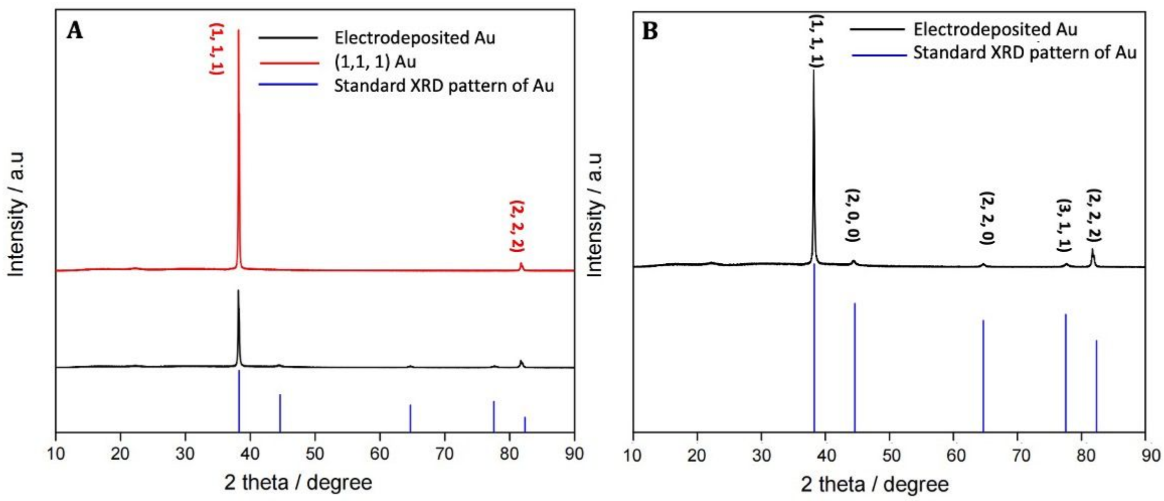

Figure S4 The XRD analysis of the bare (111) Au platform (A). and the electrodeposited Au XRD pattern (B),

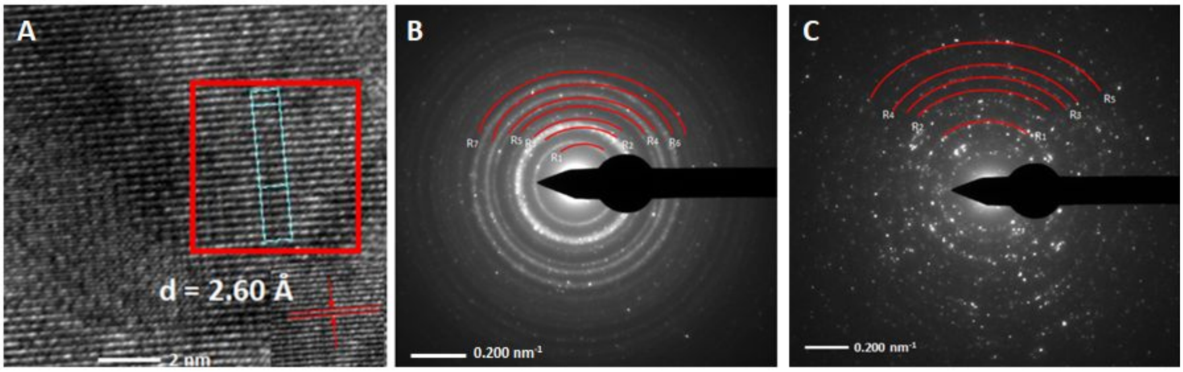

Figure S5. TEM and SAED micrographs of Rh_2O_3 NPs (A) (B) and Au (C)

Table S1. The analysis of electron diffraction patterns in Figure 1 A vs Standard d spacing of Rh_2O_3 shown in Figure S5.

Planes shown in Figure 1A	Measured d spacing (Å) in TEM	Standard d spacing (Å)	Standard 2Theta (°)
(0, -1, -1)	3.860	3.713	23.95
(-2, -1, -1)	2.813	2.713	32.99
(-2, -2, -2)	2.291	2.294	39.23
(-2, -2, 0)	1.913	1.857	49.03
(-2, -3, -1)	1.750	1.705	53.73
(0, -3, -1)	1.541	1.500	61.79

Table S2. The analysis of electron diffraction patterns in Figure S4 vs Standard d spacing of Au

Planes shown in Figure S4	Measured d spacing (Å) in TEM	Standard d spacing (Å)	Standard 2Theta (°)
(2, 0, 0)	2.153	2.030	44.60
(2, 2, 0)	1.468	1.440	64.68
(3, 1, 1)	1.261	1.230	77.55
(2, 2, 2)	1.163	1.170	82.35
(3, 3, 1)	0.9442	0.9400	110.0

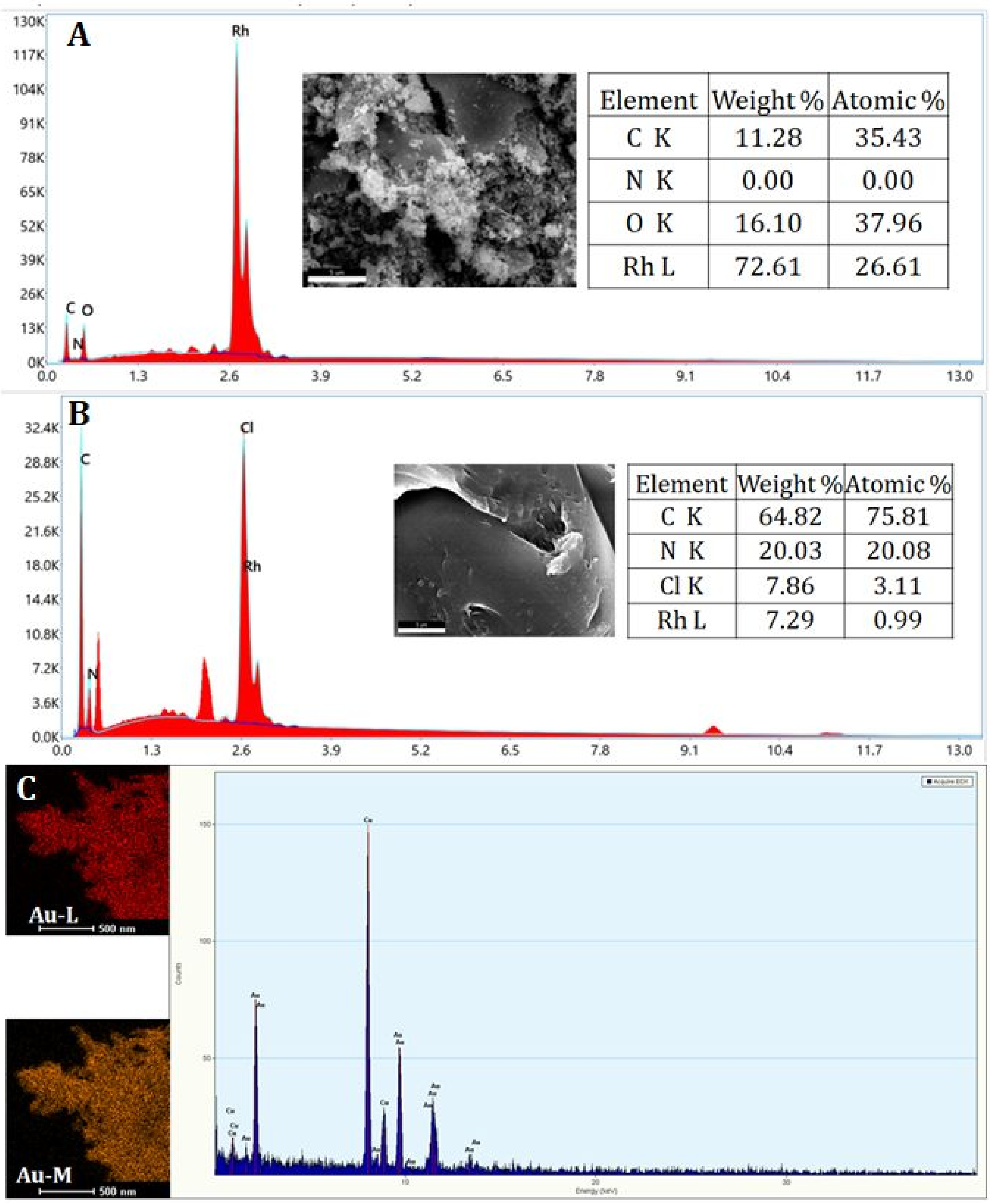

Figure S6. The EDX of calcined Rh_2O_3 (A), as-prepared $RhCl_3$/PVP precursory (B) and as-

electrodeposited Au (C)

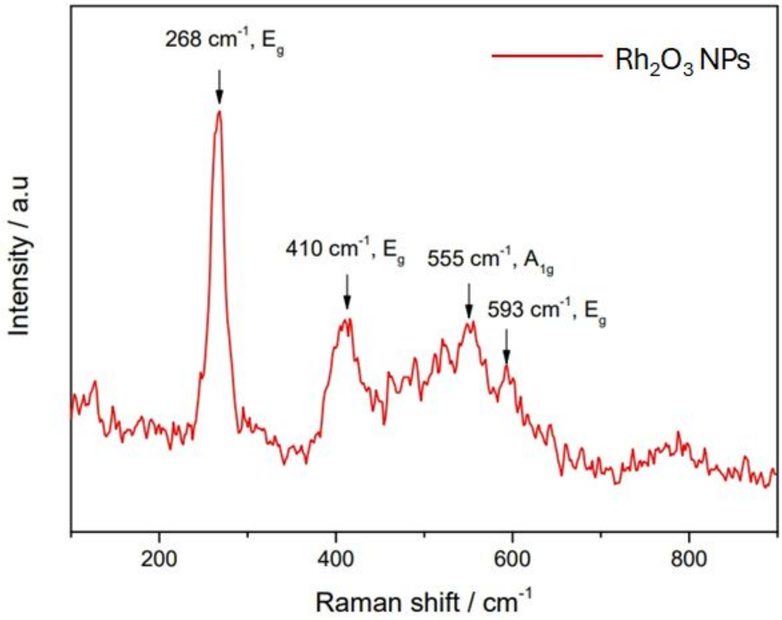

Figure S7. The Raman spectra of Rh_2O_3 NPs

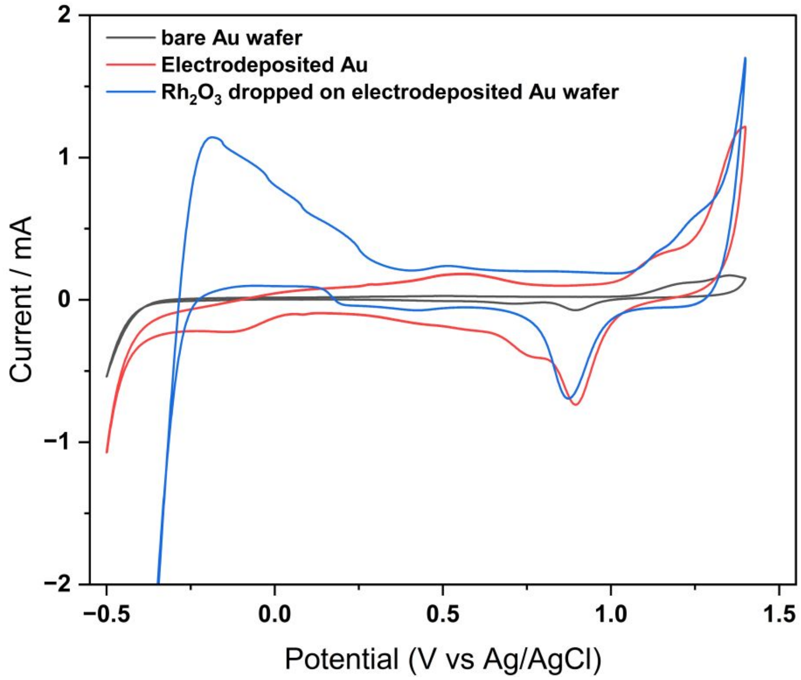

Figure S8 CVs registered in 0.1 M H_2SO_4 at 100 mV/s for Au deposited at -3.0 V for 20 s (red), mixed with Rh_2O_3 NPs (blue) and bare gold wafer (black) at a scan rate of 100 mV·s$^{-1.}$ Color should be used to indicate the differences in print.

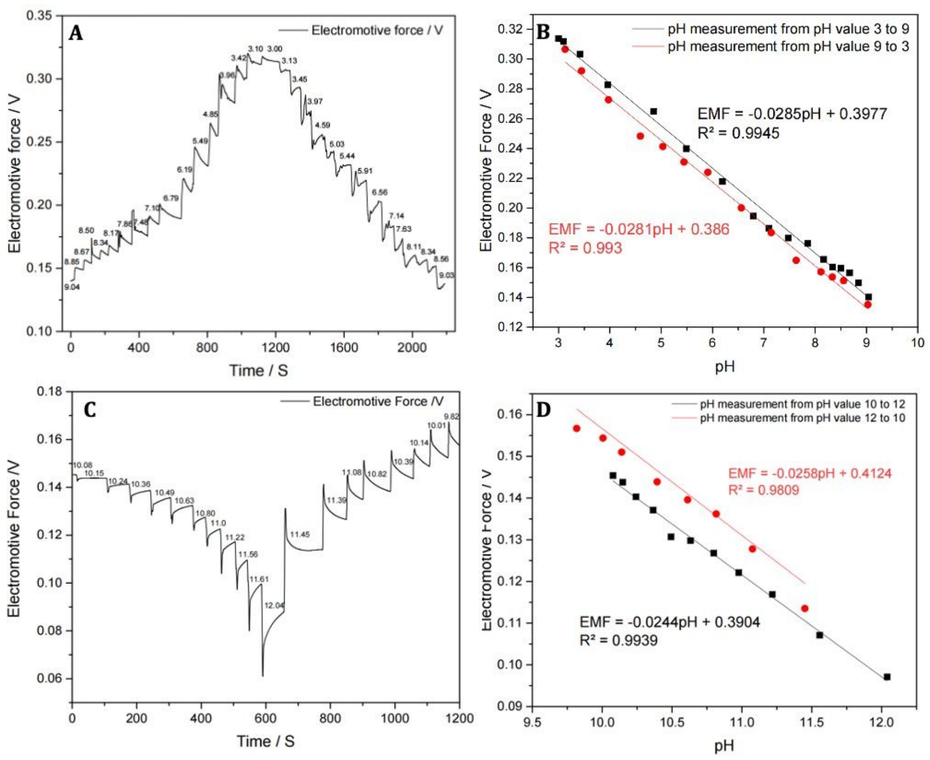

Figure S9. Electromotive force (EMF) vs. time curves for the Rh_2O_3 NPs/Nafion/GCE during reversible pH titration cycles in the range of pH 3.0–9.0 (A) and pH 10–12 (C) and their pH responses in the corresponding range of pH 3.0–9.0 (B) and pH 10–12 (D).

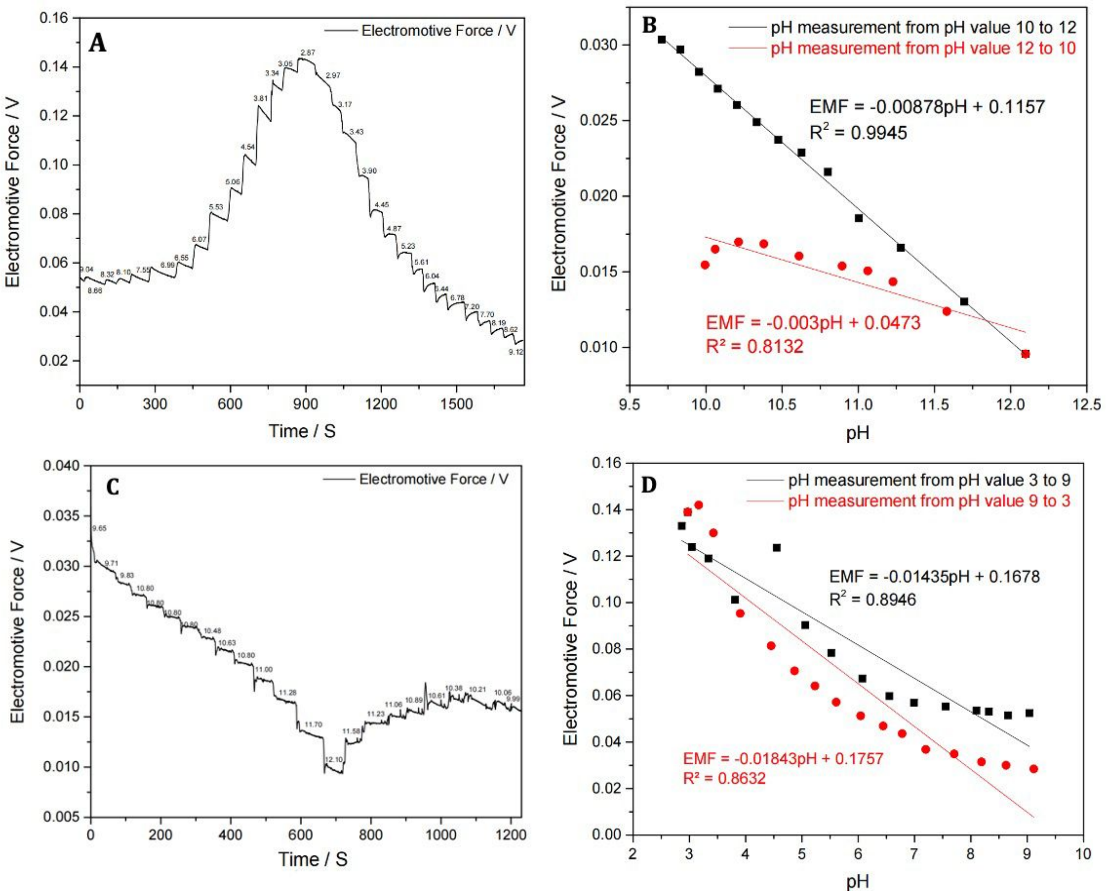

Figure S10. Electromotive force (EMF) vs. time curves for the Nafion/GCE during reversible pH titration cycles in the range of pH 3.0–9.0 (A) and pH 10–12 (C) and their pH responses in the corresponding range of pH 3.0–9.0 (B) and pH 10–12 (D).

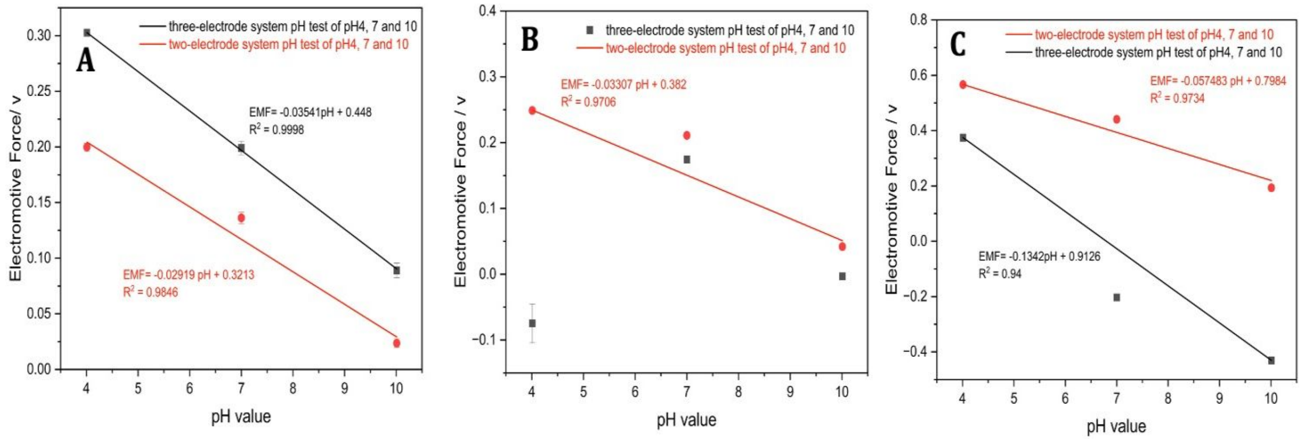

Figure S11. The pH response of different pH sensors with three types of materials (111) Au (A), nanocoral Au (B) and Rh_2O_3 dropped on nanocoral Au (C) of two-electrode and three-electrode system in standard buffer solution (4.01, 7.00, 10.01).

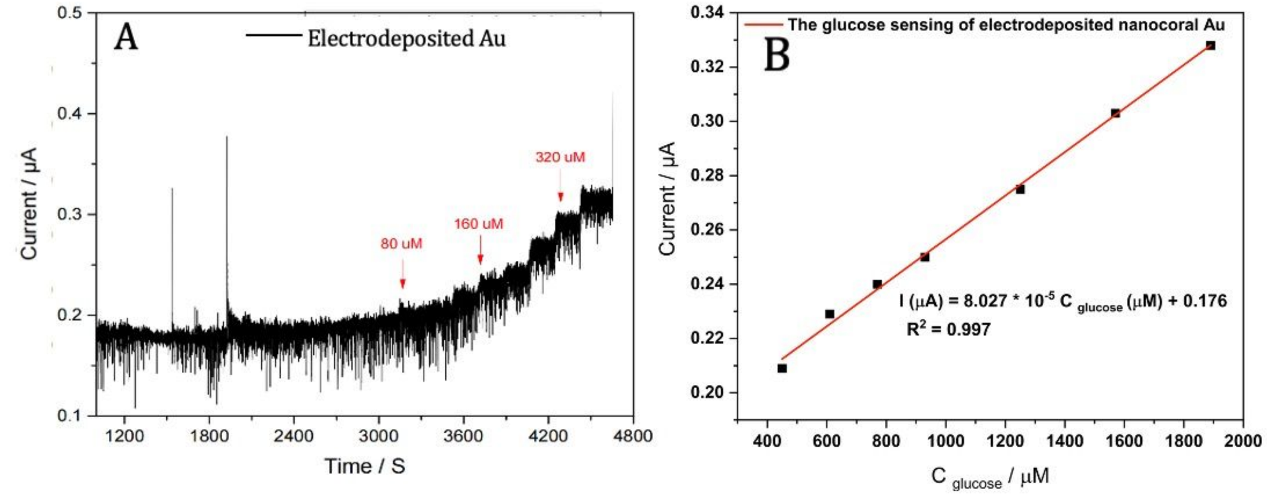

Figure S12. Amperometric response of the Electrodeposited nanocoral Au wafer (A) and the fitting curve of electrodeposited nanocoral Au wafer (red solid line). (B)

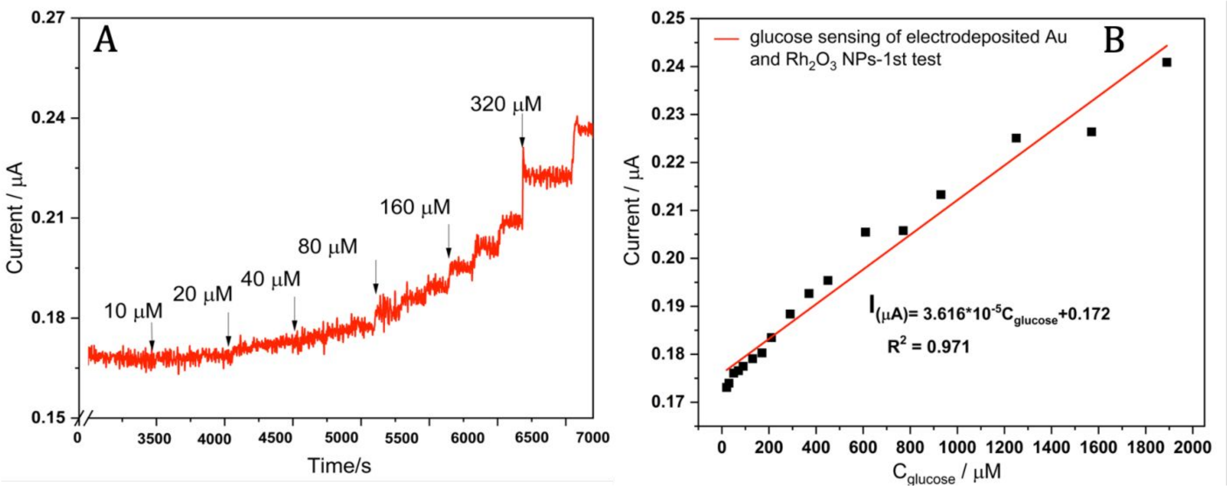

Figure S13.The 1st test of Amperometric response of Rh_2O_3 NPs doped on electrodeposited Au wafer (A) to successive addition of glucose at an applied potential of + 0.30 V (vs. Ag/AgCl) (A). and The corresponding raw data (dots) and the Langmuir isothermal fitting curve of Rh_2O_3 NPs doped on electrodeposited Au wafer (red solid line). (B)

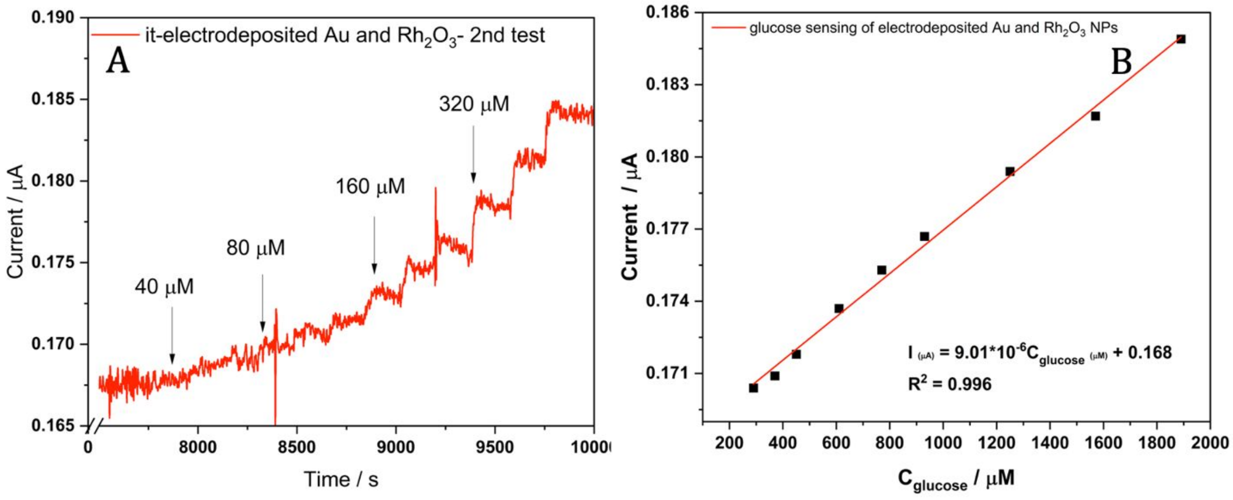

Figure S14. The 2nd test Amperometric response of one Rh_2O_3 NPs doped on electrodeposited Au wafer (A) to successive addition of glucose at an applied potential of + 0.30 V (vs. Ag/AgCl) (A), and the corresponding raw data (dots) and the Langmuir isothermal fitting curve of Rh_2O_3 NPs doped on electrodeposited Au wafer (red solid line). (B)

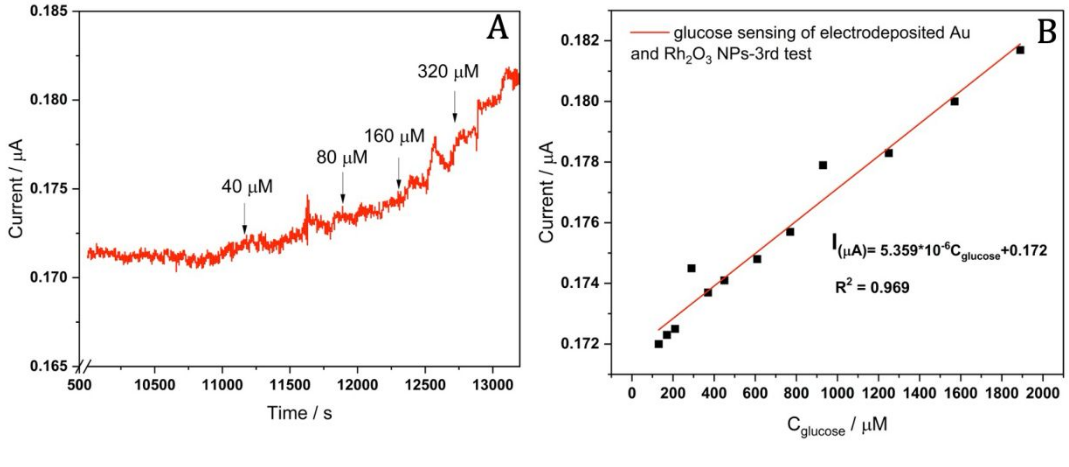

Figure S15. The 3rd test of Amperometric response of Rh_2O_3 NPs doped on electrodeposited Au wafer (A) to successive addition of glucose at an applied potential of + 0.30 V (vs. Ag/AgCl) (A). and the corresponding raw data (dots) and the Langmuir isothermal fitting curve of Rh_2O_3 NPs doped on electrodeposited Au wafer (red solid line). (B)

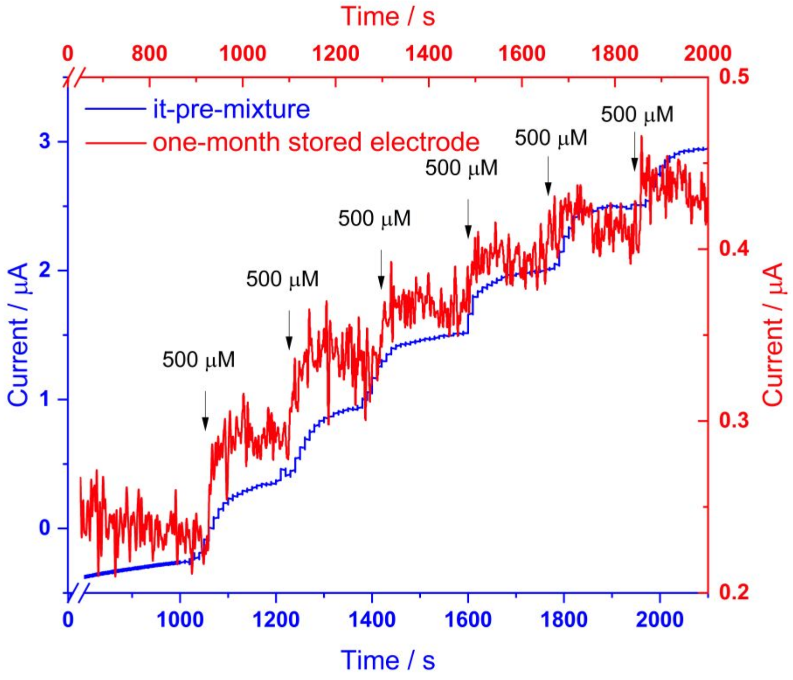

Figure S16 Amperometric response of as-prepared Rh_2O_3 NPs doped on electrodeposited Au wafer (A) and one-month storage (B) to successive addition of glucose at an applied potential of + 0.30 V (vs. Ag/AgCl). Color should be used to indicate the differences in print.

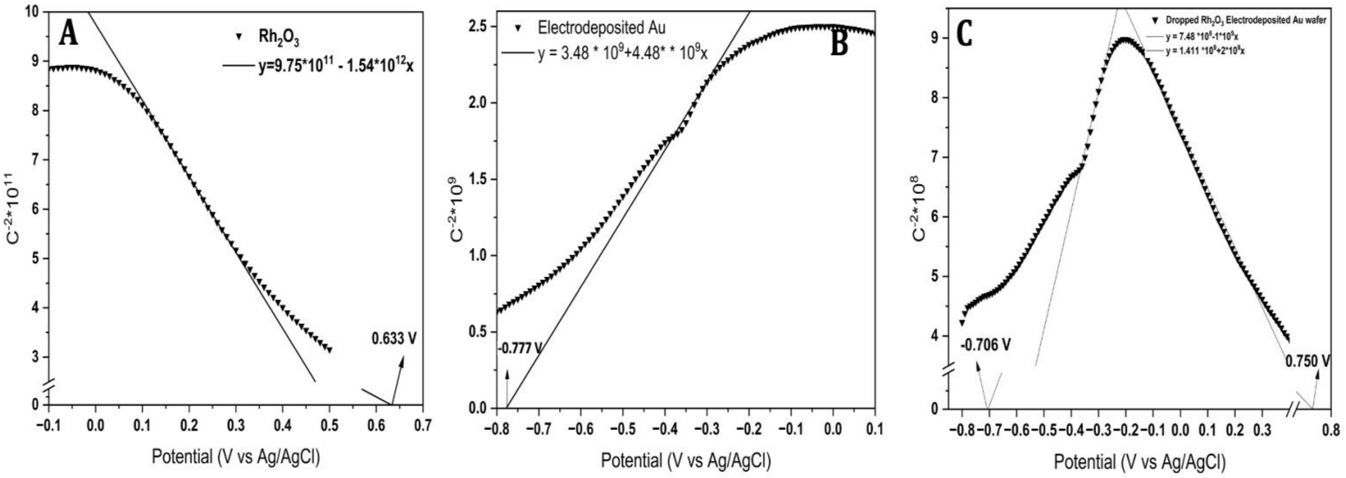

Figure S17. The Mott-Schottky plots of Rh_2O_3 NPs modified on GCE (A), nano-coral Au (B) and Rh_2O_3 NPs dropped on electrodeposited Au wafer (C).

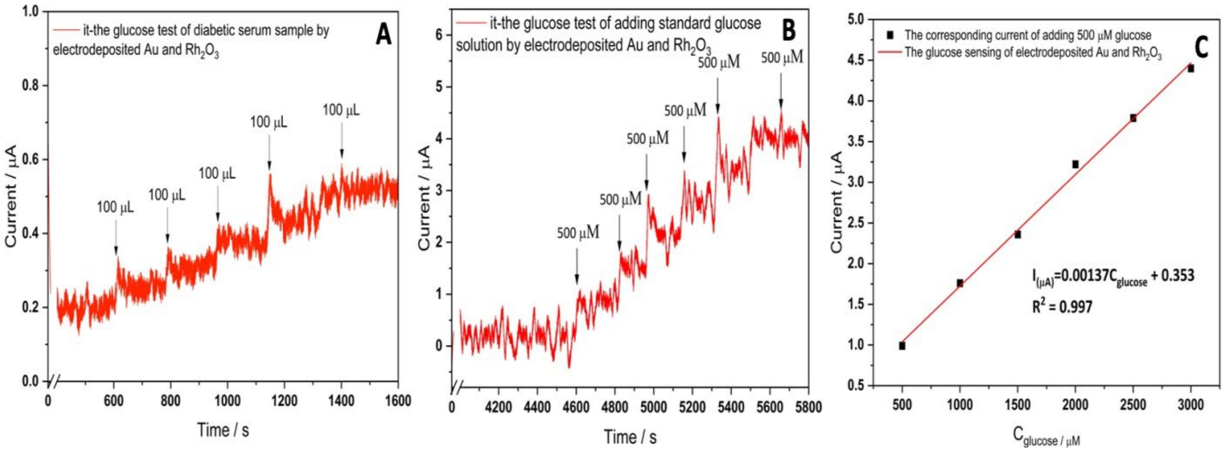

Figure S18. Amperometric response of Rh_2O_3 NPs doped on electrodeposited Au wafer to successive addition of human serum sample (A) and 100 mM standard glucose solution (B) at an applied potential of + 0.30 V (vs. Ag/AgCl) and the corresponding raw data (dots) collected in (B) and the linear fitting curve of Rh_2O_3 NPs doped on electrodeposited Au wafer (red solid line) (C).

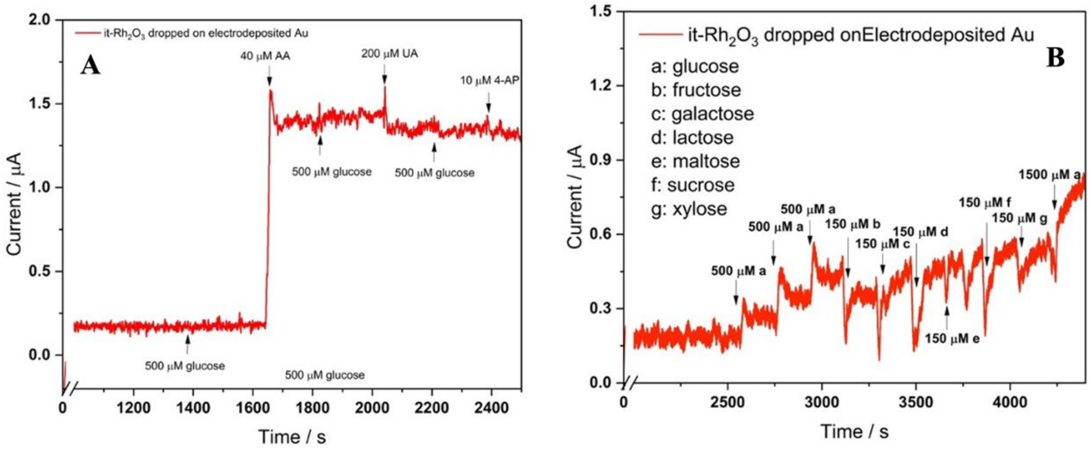

Figure S19. The amperometric response curve of the Rh_2O_3 dropped on electrodeposited Au wafer to the addition of 500 μM glucose, 10 μM 4-AP, 200 μM UA and 40 μM AA (a) and different sugars (b) in 10 mM phosphate buffer saline (pH = 7.4).

Table S3 the comparison table for the constructed glucose sensors

Sensor	Linear range	Detection of limit	Sensitivity(μA mM^{-1} cm^{-2})	Detection pH
Rh_2O_3 NPs/Nafion/GCE	Up to 1.6 mM	60 μM	20.17	pH = 13
Electrodeposited Au	-	450 μM	0.46	pH = 7.4
Rh_2O_3 doped with electrodeposited nanocoral Au	Up to 3.0 mM	20 μM	3.52	pH= 7.4

Table S4. The mean of glucose concentration determined by commercial sensor and the fabricated electrode and the t-test for a known value.

The mean of glucose concentration determined by this work / mM	The standard deviation of glucose concentration determined by this work	The mean of glucose concentration determined by commercial glucose sensor / mM	The absolute value of t
10.0	1.04	10.1	0.100
5.52	0.835	5.40	0.254
8.64	1.43	8.10	0.660
7.16	0.339	7.00	0.819
7.65	0.903	7.20	0.858
9.76	1.26	9.10	0.914
6.25	0.540	6.70	1.44
4.80	0.566	4.25	1.68
5.65	0.444	6.20	2.16
4.74	0.792	5.75	2.20
8.10	0.629	7.20	2.47
8.14	0.219	7.80	2.66
4.74	0.887	3.30	2.80
5.62	0.779	4.30	2.93
11.0	0.441	10.20	3.26
9.03	0.910	10.90	3.55
12.1	0.504	13.50	4.80
4.45	0.394	5.70	5.49
8.16	0.280	6.70	9.01
5.73	0.029	5.20	30.83
